# USP10 Is a Driver of Ubiquitinated Protein Aggregation and Aggresome Formation to Inhibit Apoptosis

**DOI:** 10.1016/j.isci.2018.11.006

**Published:** 2018-11-05

**Authors:** Masahiko Takahashi, Hiroki Kitaura, Akiyoshi Kakita, Taichi Kakihana, Yoshinori Katsuragi, Masaaki Nameta, Lu Zhang, Yuriko Iwakura, Hiroyuki Nawa, Masaya Higuchi, Masaaki Komatsu, Masahiro Fujii

**Affiliations:** 1 Division of Virology, Niigata University Graduate School of Medical and Dental Sciences, Niigata 951-8510, Japan; 2 Department of Pathology, Brain Research Institute, Niigata University, Niigata 951-8585, Japan; 3 Electron Microscope Core Facility, Niigata University, Niigata 951-8510, Japan; 4 Department of Molecular Neurobiology, Brain Research Institute, Niigata University, Niigata 951-8510, Japan; 5 Department of Microbiology, Kanazawa Medical University School of Medicine, Uchinada, 920-0293, Japan; 6 Department of Biochemistry, Niigata University Graduate School of Medical and Dental Sciences, Niigata 951-8510, Japan

**Keywords:** Molecular Mechanism of Behavior, Cellular Neuroscience, Cell Biology

## Abstract

Accumulation of ubiquitinated proteins is cytotoxic, but cells inactivate these cytotoxicities by inducing aggresome formation. We found that ubiquitin-specific protease 10 (USP10) inhibits ubiquitinated protein-induced apoptosis by inducing aggresome formation. USP10 interacted with the ubiquitin receptor p62 and the interaction augmented p62-dependent ubiquitinated protein aggregation and aggresome formation, thereby cooperatively inhibiting apoptosis. We provide evidence that USP10/p62-induced protein aggregates inhibit proteasome activity, which increases the amount of ubiquitinated proteins and promotes aggresome formation. USP10 induced aggresomes containing α-synuclein, a pathogenic protein in Parkinson disease, in cultured cells. In Parkinson disease brains, USP10 was colocalized with α-synuclein in the disease-linked aggresome-like inclusion Lewy bodies, suggesting that USP10 inhibits α-synuclein-induced neurotoxicity by promoting Lewy body formation. Collectively, these findings suggest that USP10 is a critical factor to control protein aggregation, aggresome formation, and cytotoxicity in protein-aggregation-related diseases.

## Highlights

• USP10 induces ubiquitinated protein aggregation and aggresome formation
• USP10 inhibits ubiquitinated protein-induced apoptosis by aggresome formation
• USP10 induces α-synuclein-positive aggresome
• USP10 is colocalized with α-synuclein in Lewy body in Parkinson disease

## Graphical Abstract

**Figure:**
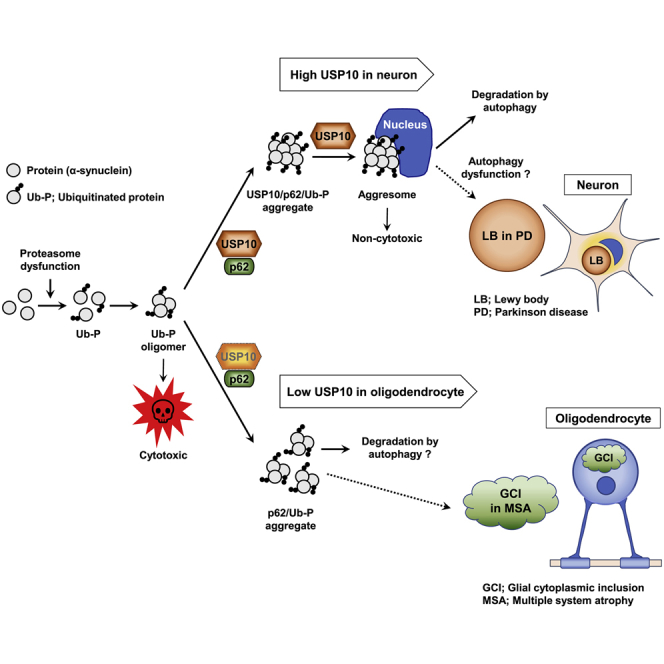

## Introduction

Various stresses such as oxidative stress generate ubiquitinated proteins with high cytotoxicity. In addition, age-related impairment of proteasome activity causes accumulation of ubiquitinated proteins, which is also associated with cell death. Aberrant accumulation of ubiquitinated proteins as inclusions is a hallmark pathology of several age-related degenerative diseases such as Parkinson disease (PD) and cystic fibrosis (Luciani et al., 2010, Ross and Poirier, 2004). Thus, cells should inactivate the cytotoxicities of ubiquitinated proteins and simultaneously reduce their amount. However, how cells inactivate the cytotoxicities of these ubiquitinated proteins and how pathogenic inclusions are formed in age-related degenerative diseases is poorly understood.

Cells activate two intracellular defense systems to inactivate the cytotoxicities of ubiquitinated proteins: the aggresome-autophagy system and the ubiquitin-proteasome system. Aggresomes are stress-inducible aggregates consisting of ubiquitinated proteins, chaperones, and proteasome components, which share many characters with pathogenic inclusions in age-related degenerative diseases (Garcia-Mata et al., 1999, Kopito, 2000, Olanow et al., 2004). p62, histone deacetylase 6 (HDAC6) and dynein play critical roles in aggresome formation. p62 is a ubiquitin receptor that interacts with ubiquitinated proteins (Katsuragi et al., 2015). Such p62-bound ubiquitinated protein aggregates interact with microtubule-associated deacetylase HDAC6, and this complex is transported to the cytoplasmic perinuclear region (microtubule-organizing center) to form aggresomes by the functions of HDAC6 and dynein motor protein in a microtubule-dependent manner (Kawaguchi et al., 2003). Aggresomes are tightly linked to selective autophagy (aggrephagy), and multiple components recruited in aggresomes are degraded by p62-dependent selective autophagy (Johansen and Lamark, 2014).

The proteasome is the main degradation machinery of ubiquitinated proteins in normal growing conditions (Tomko and Hochstrasser, 2013). Proteasome-mediated protein degradation has one crucial difference from aggrephagy. Proteasomes preferentially degrade monomeric ubiquitinated proteins, whereas aggrephagy degrades multimeric ubiquitinated protein aggregates. In addition, inhibition of proteasome activity stimulates aggresome formation (Lamark and Johansen, 2010). These results suggest that proteasome and aggresome/aggrephagy have distinct roles but coordinative regulations to inactivate the cytotoxicities of ubiquitinated proteins.

Ubiquitin-specific protease 10 (USP10) is a deubiquitinase that is ubiquitously expressed in many cell types, and a genetic knockout of *Usp10* in mice leads to bone marrow failure and death at an early age (Higuchi et al., 2016). Substrates of USP10 deubiquitinase include various stress regulators, the tumor suppressor p53 (Yuan et al., 2010), sirtuin6 (SIRT6) (Lin et al., 2013) and adenosine monophosphate-activated protein kinase (Deng et al., 2016). USP10 also has deubiquitinase-independent functions, such that USP10 inhibits apoptosis by reducing reactive oxygen species (ROS) production induced by an oxidative stress inducer arsenite (Takahashi et al., 2013b). These results suggest that USP10 is a critical stress-protective factor under various stress conditions.

In this study, we found that USP10 efficiently inactivates the cytotoxicities of ubiquitinated proteins by inducing aggresomes in a deubiquitinase-independent manner. Cystic fibrosis transmembrane conductance regulator (CFTR)-ΔF508 (Johnston et al., 1998), α-synuclein (Spillantini et al., 1997), and aminoacyl-tRNA synthetase complex-interacting multifunctional protein-2 (AIMP2) (Corti et al., 2003) are aggregation-prone proteins associated with the development of cystic fibrosis or PD. USP10 stimulated protein aggregation initiated by these proteins, thereby inducing aggresome formation. A proteasome reporter assay indicated that USP10 together with certain amounts of ubiquitination-prone proteins inhibits proteasome activity, which promoted protein aggregation and aggresome formation. To promote protein aggregation and aggresome formation, USP10 interacted with p62, and they cooperatively inhibited caspase-3-associated cell death. Importantly, USP10 was colocalized with α-synuclein of Lewy bodies in PD, and colocalization of USP10 and α-synuclein in Lewy bodies resembled those in aggresomes of cultured cells, suggesting that USP10 promotes Lewy body formation by an aggresome-related mechanism and inhibits neurotoxicities. Collectively, the present study showed that USP10 is a critical factor that inhibits cytotoxicities of ubiquitinated proteins in protein-aggregation-associated diseases by inducing aggresome formation.

## Results

### USP10 Is Localized in Aggresomes

**Figure 1:**
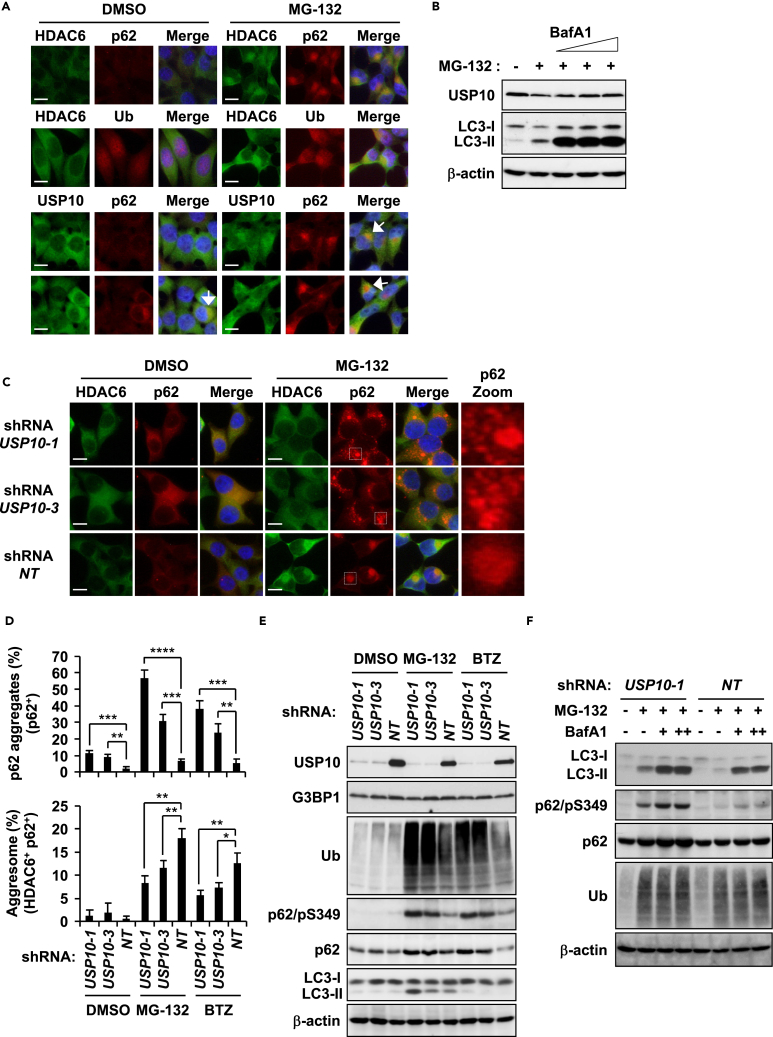
USP10 Knockdown Impairs Aggresome Formation (A) HeLa cells were treated with 5 μM MG-132 or DMSO for 12 hr, and the cells were stained with anti-HDAC6 (green) or anti-USP10 (green) antibody with either the anti-p62 (red) or anti-ubiquitin (Ub) (red) antibody. Nuclei were counterstained using Hoechst 33258 (blue). Arrows indicate cells with USP10/p62-double-positive aggregates. Scale bars, 10 μm. (B) HeLa cells were pretreated with 2.5, 5, and 10 nM bafilomycin A1 (BafA1) or DMSO for 0.5 hr and further treated with MG-132 or DMSO for 12 hr. The whole-cell extracts were characterized by western blot (WB) analysis using anti-USP10, anti-LC3, and anti-β-actin antibodies. (C) USP10-KD (*USP10-1* or *USP10-3*) and control (*NT*) HeLa cells were treated with MG-132 or DMSO for 12 hr, and the cells were stained with anti-HDAC6 (green) and anti-p62 (red) antibodies and with Hoechst 33258 (blue). Scale bars, 10 μm. (D) The indicated HeLa cells were treated with MG-132, 1 μM bortezomib (BTZ), or DMSO for 12 hr. Cells with one large HDAC6/p62-double-positive aggregate (more than 15 μm^2^ in size) at the perinuclear region with nuclear deformity were counted as aggresome-positive cells. Cells with multiple small HDAC6-negative/p62-positive aggregates (less than 10 μm^2^ in size) were counted as p62-aggregate-positive cells. The number of cells with p62 aggregates or aggresome are presented as the mean ± SD (*n* = 3); *p < 0.05; **p < 0.01; ***p < 0.001; ****p < 0.0001. (E) Whole-cell extracts prepared from the indicated HeLa cells were characterized by WB using anti-USP10, anti-G3BP1, anti-Ub, anti-p62/pS349, anti-p62, anti-LC3, and anti-β-actin antibodies. (F) The indicated HeLa cells were pretreated with increasing concentrations of BafA1 (–, DMSO; +, 5 nM BafA1; ++, 10 nM BafA1) and further treated with MG-132 or DMSO for 12 hr. The whole-cell extracts were characterized by WB using anti-LC3, anti-p62/pS349, anti-p62, anti-Ub, and anti-β-actin antibodies. See also Figure S1.

HeLa cells were treated with proteasome inhibitor (PI) MG-132 for 12 hr to examine whether USP10 is localized in aggresomes. MG-132 treatment induced mostly one large (more than 15 μm^2^ in size) aggresome per cell, which was detected with four aggresome marker proteins (p62, HDAC6, ubiquitin, and proteasome subunit α type-3 [PSMA3]) at the perinuclear regions with nuclear deformity, and the p62-positive aggresome was colocalized with USP10 (Figures 1A and S1A). In addition, MG-132 treatment of primary-neuron-enriched cells prepared from rat cortical tissues induced one large HDAC6/p62-positive aggresome with nuclear deformity, and p62-positive aggresomes colocalized with USP10 (Figure S1B). Approximately 90% of these primary cells consisted of MAP2-positive neurons (data not shown).

Aggresomes are tightly linked to lysosome-mediated degradation of aggresome-localized proteins. We examined the interaction of aggresomes with lysosomes using a lysosome marker protein, lysosome-associated membrane protein-1 (LAMP-1). One LAMP-1-positive aggregate per aggresome was always detected (Figure S1C).

We next examined whether USP10 is degraded by autophagy. Bafilomycin A1 (BafA1) is an inhibitor of autophagosome fusion with lysosomes, and the cotreatment of MG-132 with BafA1 increased the amount of the autophagy substrate LC3-II (Figure 1B). MG-132 treatment reduced the amount of USP10, and the reduction is attenuated by cotreatment with BafA1 (Figure 1B), indicating that USP10 is degraded by autophagy.

### Knockdown of USP10 Reduces Aggresome Formation and Induces Many p62-Positive Aggregates

We next examined whether USP10 plays a role in aggresome formation in HeLa cells by RNA-interference-mediated knockdown of USP10 (USP10-KD). After treatment of HeLa cells with PI, USP10-KD reduced the number of cells with p62/HDAC6-double-positive aggresomes at the perinuclear region, whereas USP10-KD induced multiple p62-positive/HDAC6-negative aggregates throughout the cytoplasm (Figures 1C and 1D). USP10-KD also reduced aggresome formation in the 293T embryonic kidney cell line (Figures S1D–S1F).

Western blot analysis showed that treatment of wild-type USP10 (USP10-WT) cells with PI increased the amounts of ubiquitinated proteins and phosphorylated p62 at Ser349 (p62/pS349) (Figure 1E). p62/pS349 increases the interaction of p62 with Keap1, an interaction that reduces ubiquitin-dependent degradation of transcription factor Nrf2 and stimulates Nrf2-dependent transcriptional activation of antioxidant genes such as NAD(P)H dehydrogenase quinone 1 (Nqo1) (Ichimura et al., 2013). MG-132-treated USP10-KD cells expressed p62, p62/pS349, ubiquitinated proteins, and LC3-II more than USP10-WT cells (Figure 1E), and the amounts of p62, p62/pS349, and LC3-II in USP10-KD cells were further augmented by cotreatment with BafA1 (Figure 1F). These results suggested that USP10-KD reduces aggresome formation but does not inhibit the autophagic degradation of p62, p62/pS349, and LC3-II.

LC3/p62 aggregates are used as markers of autophagosomes and/or autolysosomes (autophagosomes fused with lysosomes) (Pankiv et al., 2007). LC3/p62-positive aggregates were weakly detected in USP10-KD and USP10-WT cells with or without MG-132 treatment, whereas LC3/p62-positive aggregates were prominently induced by BafA1 treatment with or without MG-132 treatment (Figure S1G). These results suggested that LC3 is degraded by autophagy in both USP10-WT and USP10-KD cells with or without MG-132 treatment, and that USP10-KD does not inhibit autophagy-mediated degradation of LC3. Noteworthy, BafA1 treatment inhibited MG-132-induced aggresome formation, whereas it induced the formation of p62/LC3-double-positive/HDAC6-negative aggregates, which resembled that observed in MG-132-treated USP10-KD cells (Figure S1G). These results suggested that the fusion of p62/LC3-positive autophagosomes with lysosomes promotes aggresome formation.

### USP10 Augments Aggresome Formation by Increasing the Amount of Ubiquitinated Proteins

**Figure 2:**
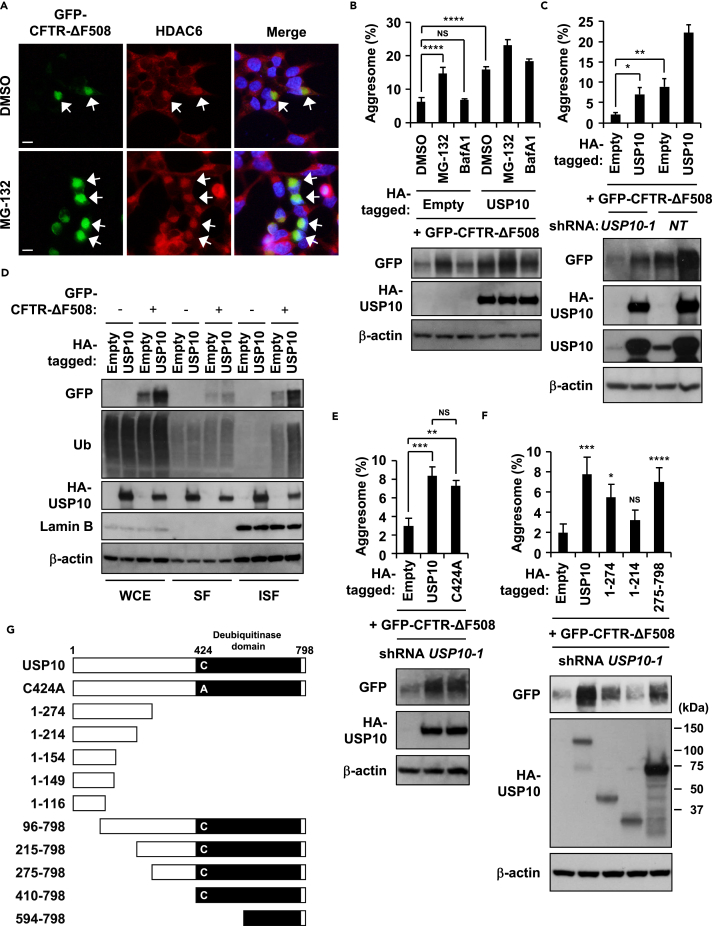
Overexpression of USP10 Stimulates Aggresome Formation (A) HeLa cells were transfected with the GFP-CFTR-ΔF508 plasmid and treated with 5 μM MG-132 or DMSO for 12 hr. The cells were stained with the anti-HDAC6 antibody (red) and Hoechst 33258 (blue). Arrows indicate localizations of HDAC6 at CFTR-ΔF508-induced aggresomes. Scale bars, 10 μm. (B) HeLa cells were transfected with the HA-tagged USP10 (HA-USP10) and GFP-CFTR-ΔF508 plasmids, and the cells were treated with MG-132, 10 nM BafA1, or DMSO for 6 hr. Cells with GFP/HDAC6-double-positive aggresomes (more than 15 μm^2^ in size) at the perinuclear region with nuclear deformity were counted as aggresome-positive cells. The percentages of cells with GFP-positive aggresome are presented as the mean ± SD (*n* = 3); ****p < 0.0001; NS, not significant. Simultaneously, whole-cell extracts were characterized by western blot (WB) using anti-GFP, anti-HA, and anti-β-actin antibodies. (C) USP10-KD (*USP10-1*) and control (*NT*) HeLa cells were transfected with the HA-USP10 and the GFP-CFTR-ΔF508 plasmids. The percentages of cells with GFP/HDAC6-positive aggresome are presented as the mean ± SD (*n* = 3); *p < 0.05; **p < 0.01. Simultaneously, whole-cell extracts were characterized by WB using anti-GFP, anti-HA, anti-USP10, and anti-β-actin antibodies. (D) HeLa cells were transfected with the HA-USP10 plasmid with or without the GFP-CFTR-ΔF508 plasmid, and the whole-cell extracts (WCE), NP-40-soluble fractions (SF), and NP-40-insoluble fractions (ISF) were characterized by WB using anti-GFP, anti-Ub, anti-HA, anti-Lamin B, and anti-β-actin antibodies. (E) USP10-KD (*USP10-1*) HeLa cells were transfected with HA-USP10 or its deubiquitinase-inactive mutant USP10^C424A^ plasmid together with the GFP-CFTR-ΔF508 plasmid. The percentages of cells with GFP/HDAC6-positive aggresome are presented as mean ± SD (*n* = 4); **p < 0.01; ***p < 0.001; NS, not significant. Simultaneously, whole-cell extracts were characterized by WB using anti-GFP, anti-HA, and anti-β-actin antibodies. (F) USP10-KD (*USP10-1*) HeLa cells were transfected with HA-USP10 or its mutant (USP10^1−274^, USP10^1−214^, or USP10^275−798^) plasmid together with the GFP-CFTR-ΔF508 plasmid. The percentages of cells with GFP/HDAC6-positive aggresome are presented as the mean ± SD (*n* = 4); *p < 0.05; ***p < 0.001; ****p < 0.0001; NS, not significant. Simultaneously, whole-cell extracts were characterized by WB using anti-GFP, anti-HA, and anti-β-actin antibodies. (G) Schematic representation of human USP10 mutants used in this study. See also Figure S2.

We next examined the activity of USP10 toward ubiquitinated protein aggregation. Cystic fibrosis is an inherited genetic disease caused by mutations of the CFTR gene (Cheng et al., 1990). CFTR-ΔF508 is a pathogenic CFTR mutant with a single amino acid deletion and is aggregation-prone (Lukacs and Verkman, 2012). Expression of GFP-tagged CFTR-ΔF508 (GFP-CFTR-ΔF508) in HeLa cells formed aggresome-like structures containing CFTR-ΔF508, and these structures were augmented by MG-132 but not BafA1, and the augmentation was accompanied by an increase in the amount of CFTR-ΔF508 (Figures 2A and 2B). Since these CFTR-ΔF508-induced aggregates were stained with aggresome marker proteins, HDAC6, p62, and ubiquitin (Figures 2A and S2A), we refer to these CFTR-ΔF508-induced aggregates as CFTR-ΔF508-induced aggresomes in the following section. ProteoStat dye visualizes misfolded protein aggregates, and staining is a marker of aggresomes (Shen et al., 2011). CFTR-ΔF508-induced aggresomes were stained with ProteoStat, suggesting that CFTR-ΔF508-induced aggresomes contain misfolded protein aggregates (Figure S2A). Coexpression of USP10 without MG-132 also promoted CFTR-ΔF508-induced aggresome formation and increased the amount of CFTR-ΔF508 (Figures 2B and S2B). In contrast, USP10-KD reduced aggresome formation of CFTR-ΔF508 and the amount of CFTR-ΔF508, and the reductions were partially rescued by exogenous USP10 (Figure 2C). Fractionation of cell lysates by a detergent showed that coexpression of USP10 with CFTR-ΔF508 predominantly increases the amounts of detergent-insoluble CFTR-ΔF508 as well as the amounts of soluble and insoluble ubiquitinated proteins (Figure 2D). These results indicate that USP10 augments CFTR-ΔF508-induced aggresome formation, and augmentation was mediated partly by increasing the amounts of CFTR-ΔF508 and ubiquitinated proteins.

To delineate the USP10 activity to aggresome formation, we characterized several USP10 mutants in USP10-KD cells. USP10^C424A^ is inactive in deubiquitinating activity (Yuan et al., 2010). We confirmed that wild-type USP10 but not USP10^C424A^ deubiquitinates the p53 protein (Figure S2C). USP10^C424A^ showed almost equivalent aggresome-augmenting activity to wild-type USP10 (USP10-WT), suggesting that the deubiquitinating activity of USP10 is not required for the aggresome-augmenting activity of USP10 (Figures 2E and S2D). USP10^1−274^ and USP10^275−798^ but not USP10^1−214^ increased the amount of CFTR-ΔF508 and simultaneously augmented aggresome formation (Figures 2F and 2G). These results suggested that both N- and C-terminal regions of USP10 are critical for maximizing aggresome-augmenting activity of USP10 to CFTR-ΔF508.

**Figure 3:**
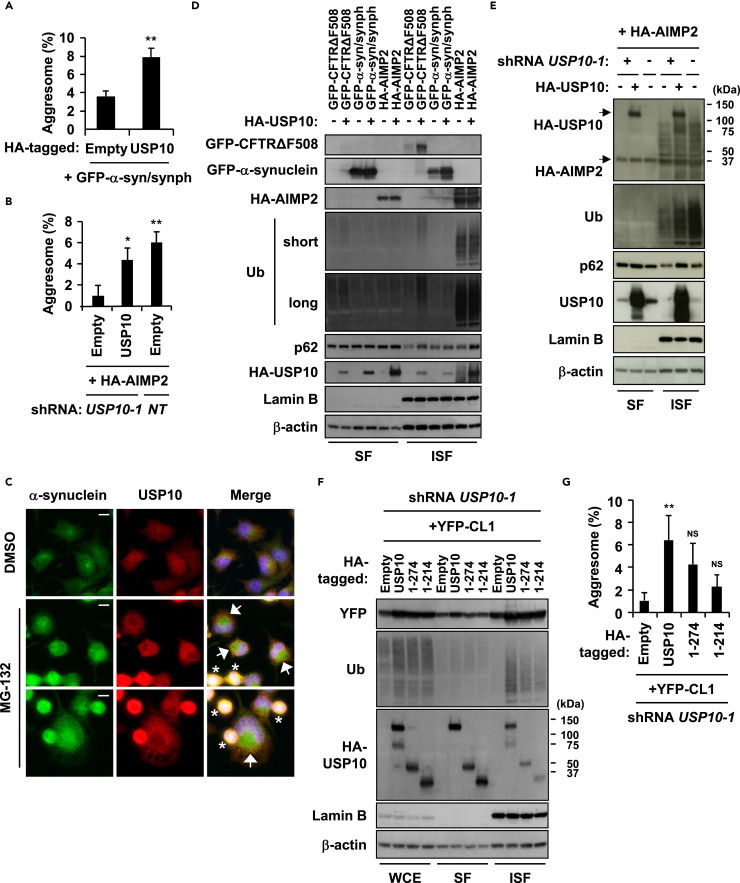
USP10 Increases the Amount of Insoluble Ubiquitinated Proteins (A) HeLa cells were transfected with HA-USP10, GFP-α-synuclein (GFP-α-syn), and Myc-synphilin-1 (synph) plasmids. The percentages of cells with GFP/USP10-positive aggresome are presented as the mean ± SD (*n* = 3); **p < 0.01. (B) USP10-KD (*USP10-1*) and control (*NT*) HeLa cells were transfected with the non-tagged USP10 and the HA-AIMP2 plasmids. The percentages of cells with HA/USP10-positive aggresomes are presented as the mean ± SD (*n* = 3); *p < 0.05; **p < 0.01. (C) Neuro-2a cells were treated with 0.5 μM MG-132 or DMSO for 12 hr. The cells were stained with the anti-α-synuclein (green) and anti-USP10 (red) antibodies and with Hoechst 33258 (blue). Arrows indicate α-synuclein-positive aggresomes induced by MG-132. Asterisks indicate dead cells. Scale bars, 10 μm. (D) HeLa cells were transfected with the HA-USP10, the GFP-CFTR-ΔF508, GFP-α-syn/synph, or the HA-AIMP2 plasmid, and NP-40-soluble fractions (SF) and NP40-insoluble fractions (ISF) were characterized by western blot (WB) using anti-GFP, anti-HA, anti-Ub, anti-p62, anti-Lamin B, and anti-β-actin antibodies. (E) USP10-KD (*USP10-1*) and control (*NT*) HeLa cells were transfected with the HA-USP10 and the HA-AIMP2 plasmids, and SF and ISF were characterized by WB using anti-HA, anti-Ub, anti-p62, anti-USP10, anti-Lamin B, and anti-β-actin antibodies. (F) USP10-KD (*USP10-1*) HeLa cells were transfected with HA-USP10 or its mutant (USP10^1−274^ or USP10^1−214^) plasmid together with the YFP-CL1 plasmid, and whole-cell extracts (WCE), SF, and ISF were characterized by WB using anti-GFP, anti-Ub, anti-HA, anti-Lamin B, and anti-β-actin antibodies. (G) The percentages of cells with YFP/HDAC6-positive aggresome are presented as the mean ± SD (*n* = 4); **p < 0.01; NS: not significant. See also Figure S3.

The generality of USP10 activity to protein aggregation was explored by examining the activity of USP10 to two additional aggregation-prone proteins, α-synuclein and AIMP2. Both α-synuclein and AIMP2 are associated with PD and are components of inclusions called Lewy bodies in brain lesions (Ko et al., 2005, Spillantini et al., 1997). Synphilin-1 is an interactor of α-synuclein, and is often coexpressed with α-synuclein to induce protein aggregation (Engelender et al., 1999). GFP-tagged α-synuclein (GFP-α-synuclein) and hemagglutinin (HA)-tagged AIMP2 (HA-AIMP2) were localized in aggresomes of HeLa cells treated with MG-132 (Figures S3A and S3B). Coexpression of USP10 with either α-synuclein or AIMP2 without MG-132 augmented the number of aggresome-like aggregates containing α-synuclein and AIMP2, respectively (Figures 3A, 3B, S3C, and S3D). USP10 was often localized at the periphery of α-synuclein- or AIMP2-induced aggresomes (Figures S3C and S3E). We also often detected localization of USP10 at the periphery of aggresomes containing endogenous α-synuclein in neuronal Neuro-2a cells (Figure 3C). Noteworthy, expression of α-synuclein without USP10 overexpression induced HDAC6/p62/ubiquitin-negative aggregates that were distinct from α-synuclein-/USP10-induced aggresome-like aggregates and these aggregates were colocalized with endogenous USP10 (Figure S3C).

Like CFTR-ΔF508, coexpression of USP10 with GFP-α-synuclein and synphilin-1 increased the amounts of insoluble α-synuclein and insoluble ubiquitinated proteins (Figure 3D). USP10 also increased the amount of non-tagged α-synuclein (Figure S3F). Coexpression of USP10 with AIMP2 slightly increased the amount of ubiquitinated proteins, but the increases were much smaller than those caused by the coexpression of USP10 with CFTR-ΔF508 (Figure 3D). As AIMP2 without coexpression of USP10 highly increased the amount of ubiquitinated proteins (Figure 3D), we assumed that endogenous USP10 is sufficient for AIMP2-mediated increase of ubiquitinated proteins. Indeed, USP10-KD dramatically reduced an AIMP2-mediated increase of ubiquitinated proteins and this reduction was rescued by the expression of exogenous USP10 (Figure 3E). These results indicated that USP10 increases the amounts of ubiquitinated proteins induced by overexpression of several aggregation-prone proteins, thereby promoting aggresome formation.

Based on USP10-induced ubiquitinated protein aggregation, we hypothesized that coexpression of USP10 with an aggregation-prone protein inhibits proteasome activity. To examine this possibility, we measured proteasome activity by using CL1. CL1 is the 16-amino-acid polypeptide containing an ubiquitination-prone site (Gilon et al., 1998). YFP-CL1 is a fusion protein of CL1 with yellow fluorescent protein (YFP), and it is degraded by proteasomes. Thus, YFP-CL1 is used as a reporter to measure proteasome activity (Menendez-Benito et al., 2005). Coexpression of USP10 with YFP-CL1 increased the amounts of insoluble YFP-CL1 and insoluble ubiquitinated proteins (Figure 3F). These results suggested that coexpression of USP10 with an aggregation-prone protein inhibits proteasome activity to increase the amount of insoluble ubiquitinated proteins, thereby augmenting aggresome formation. Like CFTR-ΔF508, the aggregation-inducing activities of USP10-WT toward YFP-CL1 was higher than those of USP10^1−274^ and USP10^1−214^ (Figure 3G).

### USP10 Promotes Aggresome Formation by Interacting with p62

**Figure 4:**
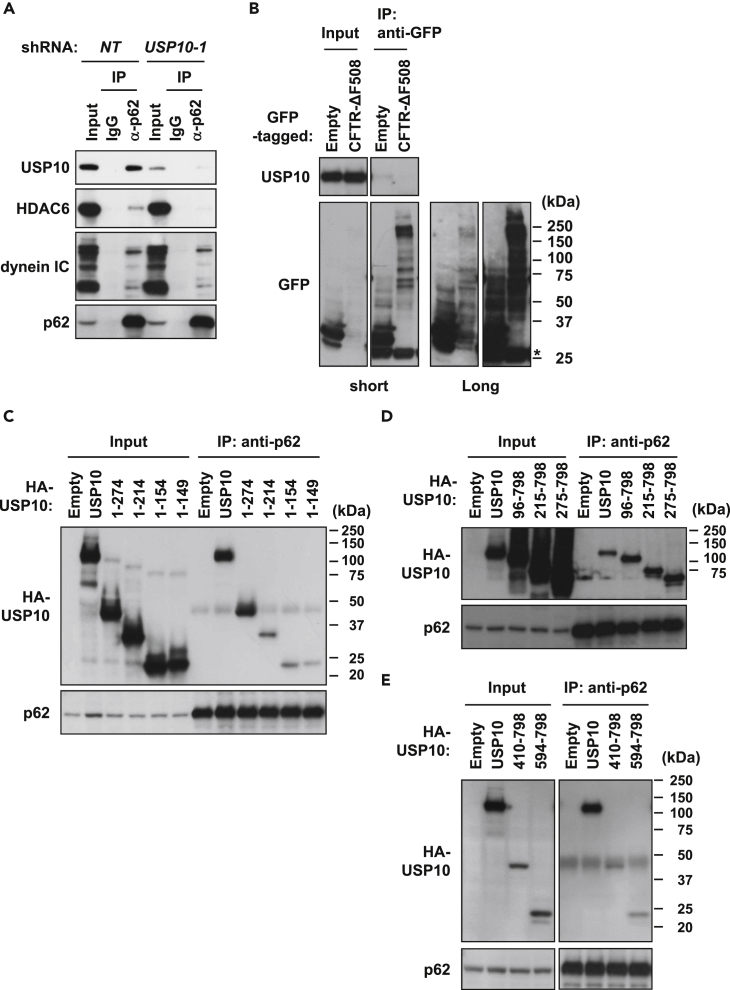
p62 Interacts with USP10 (A) Cell lysates prepared from control (*NT*) and USP10-KD (*USP10-1*) HeLa cells were immunoprecipitated with the anti-p62 antibody or normal rabbit IgG. The cell lysate (Input) and immunoprecipitates (IP) were characterized by western blot (WB) with anti-USP10, anti-HDAC6, anti-dynein intermediate chain (IC), and anti-p62 antibodies. (B) HeLa cells were transfected with the GFP-CFTR-ΔF508 plasmid or empty control. Cell lysates (Input) and immunoprecipitates with the anti-GFP antibody (IP) were characterized by WB using anti-USP10 and anti-GFP antibodies. The asterisk indicates a nonspecific band. (C–E) HeLa cells were transfected with HA-USP10 or its mutant (USP10^1−274^, USP10^1−214^, USP10^1−154^, or USP10^1−149^ in C; USP10^96−798^, USP10^215−798^, or USP10^275−798^ in D; and USP10^410−798^ or USP10^594−798^ in E) plasmid. Cell lysates (Input) and immunoprecipitates with the anti-p62 antibody (IP) were characterized by WB with anti-HA and anti-p62 antibodies.

p62 is a ubiquitin-binding protein and plays a critical role in ubiquitinated protein aggregation (Komatsu et al., 2007). Thus, we examined whether USP10 interacts with p62. The immunoprecipitation assay indicated that endogenous p62 interacted with endogenous USP10 together with previously known p62 interactors HDAC6 or dynein (Calderilla-Barbosa et al., 2014, Yan et al., 2013), both of which are important for aggresome formation (Figure 4A). In contrast, the interaction of USP10 with CFTR-ΔF508 could not be detected (Figure 4B). Co-immunoprecipitation of USP10 deletion mutants with p62 indicated that both N- and C-terminal regions of USP10, USP10^1−274^, and USP10^275−798^, but not USP10^1−214^, interact with p62 (Figures 4C–4E and 2G). As USP10^1−274^ and USP10^275−798^, but not USP10^1−214^, promoted aggresome formation induced by CFTR-ΔF508 (Figure 2F), these results suggested that USP10 promotes aggresome formation by interacting with p62.

**Figure 5:**
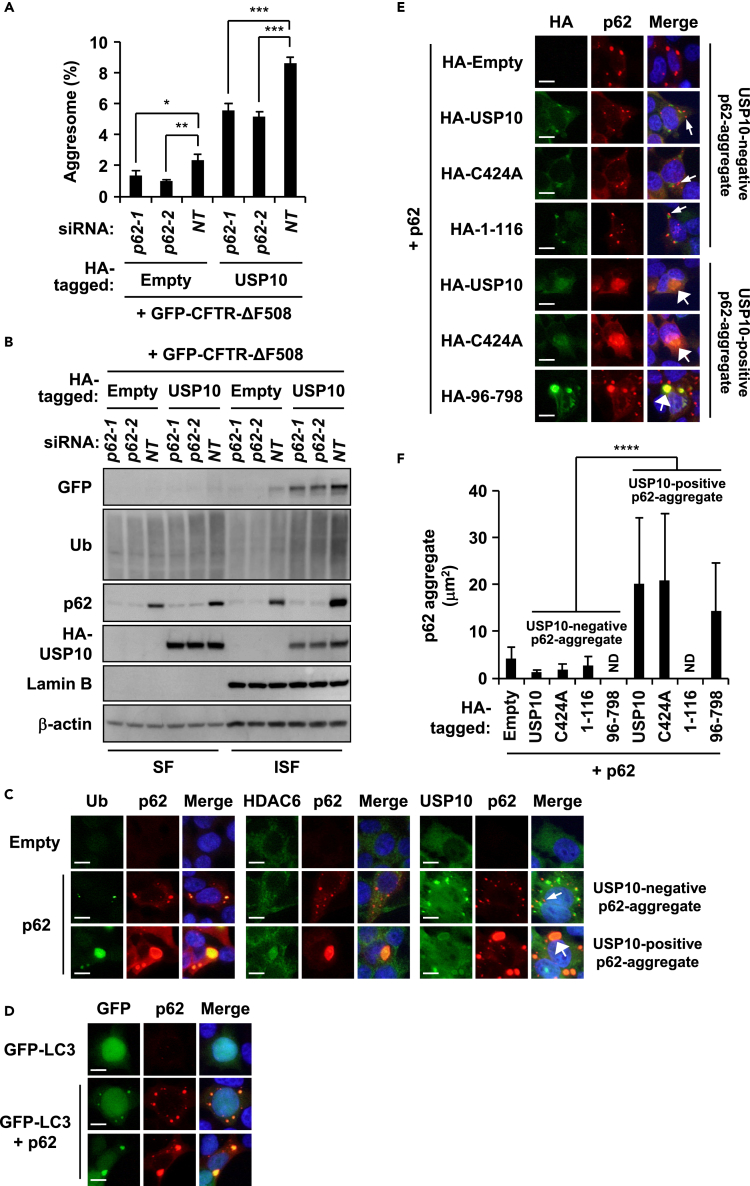
p62 Mediates USP10-Induced Protein Aggregation (A) HeLa cells were transfected with p62 small interfering RNA (siRNA) (*p62-1* or *p62-2*) or control siRNA (*NT*) and were further transfected with the HA-USP10 and the GFP-CFTR-ΔF508 plasmids. The percentages of cells with GFP/HDAC6-positive aggresome are presented as the mean ± SD (*n* = 4); *p < 0.05; **p < 0.01; ***p < 0.001. (B) The NP-40-soluble fractions (SF) and NP-40-insoluble fractions (ISF) from the indicated HeLa cells were characterized by western blot (WB) using anti-GFP, anti-Ub, anti-p62, anti-HA, anti-Lamin B, and anti-β-actin antibodies. (C) HeLa cells were transfected with the p62 plasmid. The cells were stained with the anti-p62 (red) antibody and with anti-Ub (green), anti-HDAC6 (green), or anti-USP10 (green) antibody, and with Hoechst 33258 (blue). The small arrow indicates USP10-negative p62 aggregate, whereas the large arrow indicates USP10-positive p62 aggregate. Scale bars, 10 μm. (D) HeLa cells were transfected with the p62 and the GFP-LC3 plasmids. The cells were stained with the anti-p62 antibody (red) and Hoechst 33258 (blue). Scale bars, 10 μm. (E) HeLa cells were transfected with the p62 plasmid and HA-USP10 or its mutant plasmids (USP10^C424A^, USP10^1−116^, or USP10^96−798^). The cells were stained with anti-HA (green) and anti-p62 (red) antibodies, and with Hoechst 33258 (blue). Small arrows indicate HA-USP10-negative p62 aggregates, whereas the large arrows indicate HA-USP10-positive p62 aggregates. Scale bars, 10 μm. (F) The sizes of p62 aggregates (μm^2^) are presented as the mean ± SD (*n* = 30); ****p < 0.0001; ND, not detected. See also Figure S4.

To investigate whether p62 plays a positive role in USP10-induced protein aggregation, we examined CFTR-ΔF508-induced protein aggregation in p62-KD cells. p62-KD reduced the amounts of insoluble CFTR-ΔF508, insoluble ubiquitinated proteins, and insoluble USP10 induced by CFTR-ΔF508/USP10, and simultaneously reduced aggresome formation (Figures 5A and 5B). Moreover, USP10 expression increased the amount of insoluble p62 in p62 wild-type cells (Figure 5B). Collectively, these results indicated that USP10 induces ubiquitinated protein aggregation and aggresome formation, and this is facilitated partly by interacting with p62.

Conversely, we examined the role of USP10 in p62-induced protein aggregation. Overexpression of p62 in HeLa cells without MG-132-treatment induced large and small p62-positive aggregates (Figure 5C). Large p62-positive aggregates were colocalized with ubiquitin, HDAC6, USP10, and GFP-LC3 (LC3 fusion protein of GFP), and therefore resembled aggresomes (Figures 5C and 5D). In contrast, small p62 aggregates were colocalized with ubiquitin and GFP-LC3, but not with USP10 (Figures 5C and 5D), and these aggregates resembled p62 aggregates in USP10-KD cells treated with MG-132 (Figure 1C). Coexpression of USP10 with p62 augmented the sizes of p62-induced aggregates/aggresomes (Figures 5E and 5F). These results suggested that USP10 converts small USP10-negative p62 aggregates to USP10/p62-double-positive aggregates/aggresomes. In addition to USP10-WT, USP10^96−798^, but not USP10^1−116^, increased the size of p62 aggregates/aggresomes (Figures 5E and 5F). Given that USP10^96−798^ but not USP10^1−214^ interacts with p62 (Figures 4C and 4D), these results also suggested that USP10 interaction with p62 induces large p62 aggregates/aggresomes. USP10^C424A^ increased the size of p62-induced aggresomes equivalently to USP10-WT (Figures 5E and 5F), suggesting that the deubiquitinating activity of USP10 is not required for the augmentation of p62-induced aggregation.

USP10 showed two opposite activities to the amounts of p62 and ubiquitinated proteins in cells either treated or not treated with MG-132 (Figures 5B and 1E). USP10 overexpression without MG-132 treatment increased the amounts of p62 and insoluble ubiquitinated proteins (Figure 5B), whereas USP10-KD cells treated with MG-132 increased the amounts of p62 and ubiquitinated proteins more than USP10-WT cells (Figure 1E). We showed previously that USP10-KD increases the production of ROS by an oxidant (arsenite) (Takahashi et al., 2013a). The different USP10 activities might be explained by the production of ROS stimulated by MG-132 treatment. Since ROS increases the expression of p62 by activating transcription factor Nrf2 (Jain et al., 2010, Jaramillo and Zhang, 2013), ROS produced in MG-132-treated USP10-KD cells might induce p62 and ubiquitinated proteins more than USP10-WT cells. USP10-KD cells treated with MG-132 possessed more ROS and nuclear Nrf2 than USP10-WT cells, and cells with high nuclear Nrf2 induced more p62 protein (Figures S4A–S4C). In addition, an antioxidant N-acetylcysteine reduced the amount of p62 aggregates in USP10-KD cells treated with PI (Figure S4D). These results suggested that MG-132 treatment stimulates ROS production more in USP10-KD cells than in USP10-WT cells, and such ROS then induce nuclear activated Nrf2 in USP10-KD cells, thereby increasing the amounts of p62 and ubiquitinated proteins. These results suggested that USP10 has two opposing activities to p62-dependent protein aggregation that are dependent on the level of ROS.

### USP10 Inhibits Cell Death Induced by MG-132

**Figure 6:**
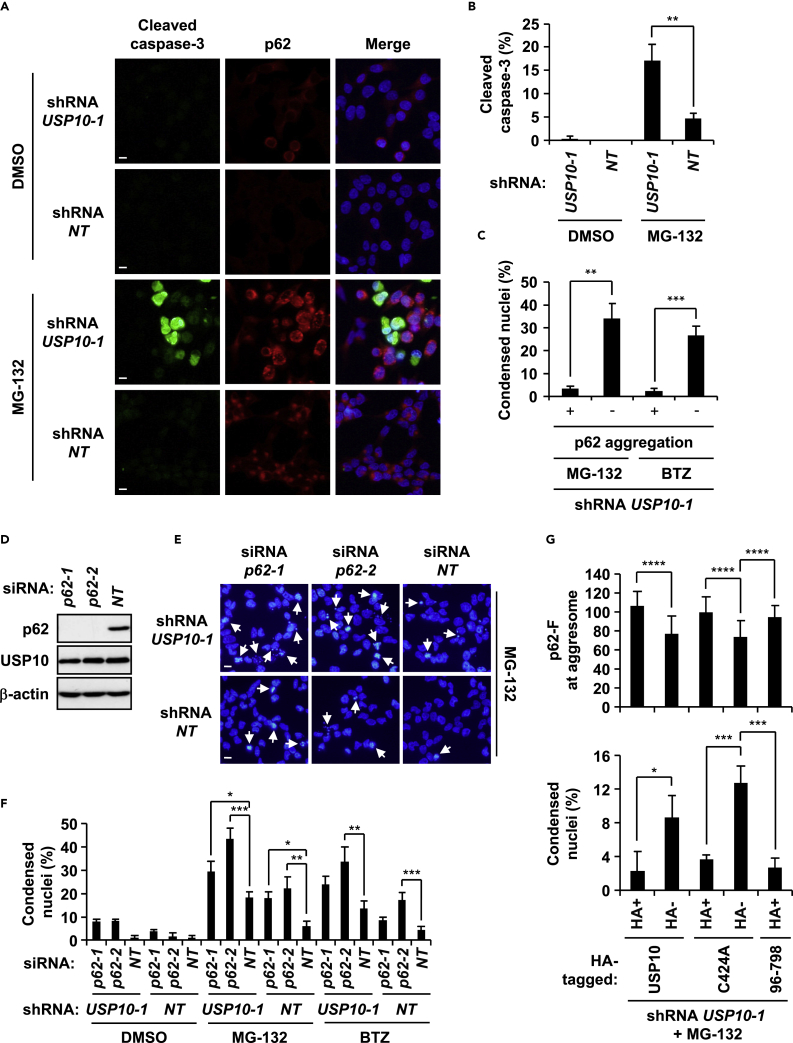
USP10 Knockdown Augments Cell Death Induced by a Proteasome Inhibitor (A) USP10-KD (*USP10-1*) and control (*NT*) HeLa cells were treated with 5 μM MG-132 or DMSO for 12 hr, and the cells were stained with anti-cleaved caspase-3 (green) and anti-p62 (red) antibodies, and with Hoechst 33258 (blue). Scale bars, 10 μm. (B) Apoptotic cells were assessed by staining cleaved caspase-3. Proportions of cleaved caspase-3-positive cells in USP10-KD (*USP10-1*) and control (*NT*) HeLa cells are presented as the mean ± SD (*n* = 3); **p < 0.01. (C) USP10-KD (*USP10-1*) HeLa cells were treated with MG-132 or 1 μM bortezomib (BTZ) for 12 hr, and the cells were stained with the anti-p62 antibody and Hoechst 33258. Proportions of cells containing condensed nuclei with or without p62 aggregates are presented as the mean ± SD (*n* = 3); **p < 0.01; ***p < 0.001. (D) HeLa cells were transfected with p62 small interfering RNA (siRNA) (*p62-1* or *p62-2*) or control siRNA (*NT*) and cultured for 48 hr. Whole-cell extracts were characterized using western blot with anti-p62, anti-USP10, and anti-β-actin antibodies. (E) USP10-KD (*USP10-1*) and control (*NT*) HeLa cells were transfected with p62 siRNA (*p62-1* or *p62-2*) or control siRNA (*NT*), and further treated with MG-132 for 12 hr. Cells were stained with Hoechst 33258 (blue). The arrows indicate cells containing condensed nuclei (apoptotic cells). Scale bars, 10 μm. (F) Proportions of cells containing condensed nuclei (apoptotic cells) are presented as the mean ± SD (*n* = 3); *p < 0.05; **p < 0.01; ***p < 0.001. (G) p62 fluorescence at aggresome (more than 15 μm^2^ in size) (p62-F at aggresome; *n* = 40) or the proportions of condensed nuclei (condensed nuclei [%]; *n* = 3) in USP10-KD (*USP10-1*) HeLa cells expressing wild-type USP10, USP10^C424A^, or USP10^96−798^ from Figures S5A or S5B are presented as the mean ± SD; *p < 0.05; ***p < 0.001; ****p < 0.0001. See also Figure S5.

MG-132 induces cell death of cultured cells, and the death of these cells is inhibited by aggresome formation (Kawaguchi et al., 2003, Tanaka et al., 2004). Thus, we examined whether USP10 inhibits MG-132-induced cell death by measuring activated caspase-3 (cleaved caspase-3). An anti-cleaved caspase-3 antibody detected cell death of USP10-KD cells treated with MG-132, and the level was more than that of USP10-WT cells (Figures 6A and 6B). Interestingly, USP10-KD cells with p62 aggregates were more resistant to PI-induced cell death than cells without p62 aggregates, suggesting that p62 aggregates inhibit cell death induced by PI (Figure 6C).

To further examine the role of p62 in PI-induced cell death, we examined the sensitivity of p62-KD cells to PI. Nuclear condensation analysis showed that MG-132-induced cell death was augmented by either p62-KD or USP10-KD, and the level was further increased by their double-knockdowns (Figures 6D–6F). These results indicated that USP10 and p62 cooperatively inhibit MG-132-induced cell death by promoting the formation of aggresomes and p62 aggregates.

To obtain information describing how USP10 inhibits MG-132-induced cell death, we measured cell death of USP10-KD cells expressing several USP10 mutants. USP10-KD cells expressing USP10-WT were resistant to cell death induced by MG-132 more than cells without USP10-WT (Figures 6G, S5A, and S5B). USP10^C424A^ and USP10^96−798^, but not USP10^1−214^, reduced cell death of USP10-KD cells (Figures 6G and S5A–S5C). Given that USP10-WT, USP10^C424A^, and USP10^96−798^, but not USP10^1−214^, in USP10-KD cells promoted p62 aggregation and aggresome formation (Figures 6G and S5A–S5C), these results suggested that USP10 inhibits cell death induced by MG-132 by promoting aggresome formation and p62 aggregation.

**Figure 7:**
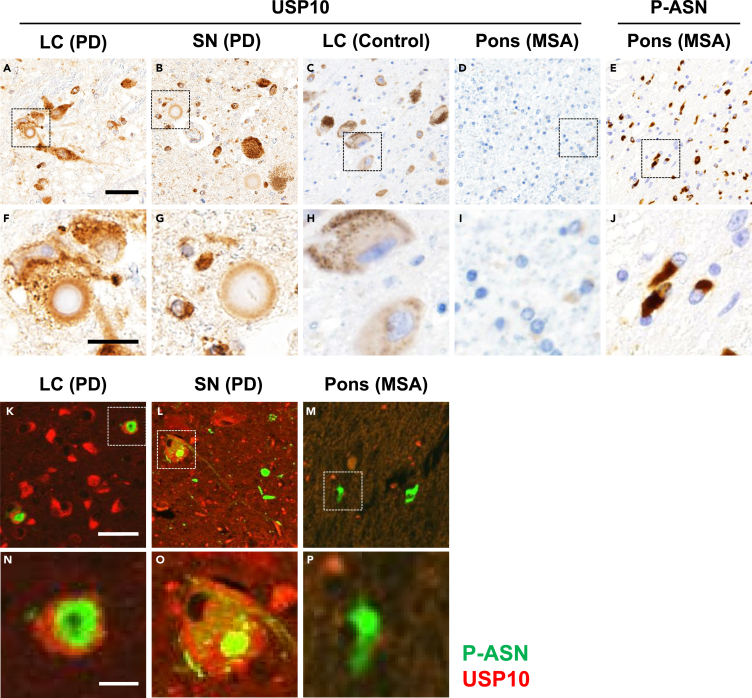
Localizations of USP10 and α-Synuclein in Brain (A–J) Representative images taken from USP10- or phosphorylated α-synuclein (P-ASN)-immunostained sections of patients with PD (PD, *n* = 3), patients with MSA (MSA, *n* = 3), and controls (*n* = 3). (A–D) and (F–I) USP10 staining; (E and J) P-ASN staining. (F–J) The magnified images of (A–E). (A and F) Locus coeruleus (LC) of PD; (B and G) substantia nigra (SN) of PD; (C and H) LC of control; (D, E, I, and J) pons of MSA. (K–P) Images of double-immunofluorescence staining of USP10 and P-ASN. (N–P): magnified images of (K–M). (K and N) LC of PD; (L and O) SN of PD; (M and P) pons of MSA. Scale bars, 50 μm in (A–E and K–M) and 10 μm in (F–J and N–P). See also Table S1.

PD, a representative neurodegenerative disorder of synucleinopathy, always bears many α-synuclein-positive neuronal inclusions, namely, Lewy bodies (Peng et al., 2017). We found characteristic USP10 immunoreactivity in Lewy bodies in neurons of the substantia nigra, locus coeruleus, and other brain stem nuclei, where the halo and core of the inclusions were positive and negative, respectively (Figures 7A, 7B, 7F, and 7G and Table S1). A double-labeling immunofluorescent study demonstrated colocalization of phosphorylated α-synuclein and USP10 in Lewy bodies (Figures 7K, 7L, 7N, and 7O), indicating the possible association of both proteins. In contrast, in neurons of the controls and patients with PD without Lewy bodies, the reactivity was diffuse within the cytoplasm (Figures 7C and 7H). These results suggested that USP10 is involved in Lewy body formation.

We also examined USP10 immunoreactivity for oligodendroglial cytoplasmic inclusions (GCIs) in multiple system atrophy (MSA) (Figures 7D and 7I and Table S1), another representative disorder of synucleinopathy (Ahmed et al., 2012). We found no reactivity of anti-USP10 toward GCIs and oligodendrocytes in MSA and control samples (Figures 7D and 7I). Therefore, involvement of USP10 seems characteristic for Lewy bodies, rather than a common phenomenon in α-synuclein-containing inclusions.

**Figure 8:**
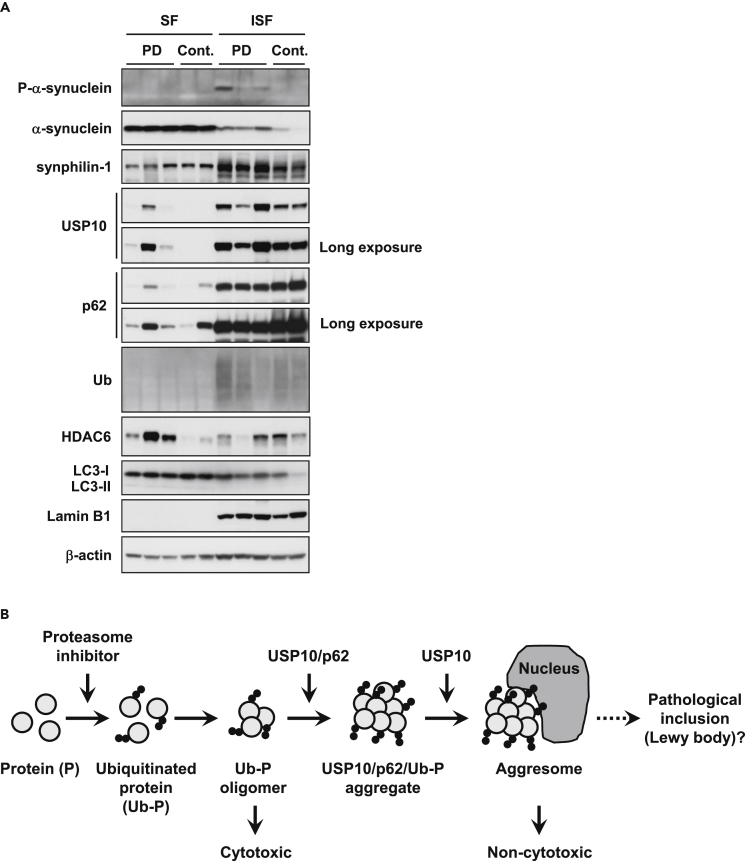
Expression Levels of USP10 and Aggresome-Related Proteins in Brain Lesions of Patients with PD (A) Triton X-100-soluble fractions (SF) and Triton X-100-insoluble fractions (ISF) were prepared from brain tissues (amygdala) of three patients with PD (PD) and two controls (Cont.), and the lysates were characterized by western blot using anti-phosphorylated α-synuclein, anti-α-synuclein, anti-synphilin-1, anti-USP10, anti-p62, anti-Ub, anti-HDAC6, anti-LC3, anti-Lamin B1, and anti-β-actin antibodies. See also Table S2. (B) A current model describing the function of USP10 on protein aggregation.

Finally, we measured the amounts of α-synuclein, USP10, and aggresome-related proteins in brain (amygdala) lesions of patients with PD after separating the samples into Triton X-100-soluble and Triton X-100-insoluble fractions (Figure 8A and Table S2). Western blot analysis showed that the three patients with PD expressed more phosphorylated α-synuclein, α-synuclein, and synphilin-1 in the detergent-insoluble fraction than the two control samples. These results are consistent with the fact that phosphorylated α-synuclein is the major component of Lewy bodies in patients with PD (Anderson et al., 2006). Patients with PD also expressed soluble and/or insoluble USP10 proteins more than the controls. In addition, the amount of aggresome-inducing protein HDAC6 in the soluble fraction was increased in PD samples relative to the control samples. These results support the notion that USP10 and aggresomes play a role in Lewy body formation (Figure 8B).

## Discussion

Ubiquitinated proteins generated under various stress conditions, such as oxidative stress and proteasome dysfunction, are cytotoxic. To inactivate these cytotoxicities, cells induce aggresomes to promote cell survival. In this study, we found that USP10 is an essential factor to inactivate cytotoxicities of ubiquitinated proteins by inducing aggresome formation.

We showed that USP10 promotes aggresome formation partly by inhibiting the proteasome-mediated degradation of ubiquitinated proteins. The CFTR-ΔF508 protein was degraded by proteasomes in HeLa cells (Figure 2B), and coexpression with USP10 increased the amount of insoluble ubiquitinated proteins, including CFTR-ΔF508 by itself, which promoted aggresome formation (Figures 2B and 2D). Such USP10 activity was also detected with two other ubiquitination-prone proteins, α-synuclein and AIMP2 (Figures 3A, 3B, 3D, and 3E). Moreover, a proteasome activity reporter YFP-CL1 indicated that USP10 together with YFP-CL1 inhibits proteasome activity (Figure 3F). These results are consistent with previous studies showing that protein aggregates inhibit proteasome activity (Hipp et al., 2014). The proteasome enzyme subunit PSMA3 was also detected in both MG-132-induced and USP10-induced aggresomes (Figures S1A and S2E). Moreover, p62 has been shown to interact with ubiquitinated proteasome components (Cohen-Kaplan et al., 2016). Thus, USP10 and p62 might inhibit proteasome activity by trapping proteasome components in protein aggregates and/or aggresomes.

Interaction of USP10 with p62 promoted protein aggregation and aggresome formation (Figure 5). Analysis using USP10 deletion mutants showed that both N- and C-terminal regions of USP10 interact with p62 and that these two regions are required for maximum aggresome-promoting activity (Figure 2F). These results suggested that multiple interactions between USP10 and p62 are required for maximum aggresome-promoting activity of USP10. It should be noted that p62-KD reduced protein aggregation and aggresome formation induced by USP10/CFTR-ΔF508, and the reduction was approximately half of the p62-competent cells (Figures 5A and 5B). Since several ubiquitin receptors other than p62, such as optineurin, neighbor of BRCA1 gene 1 (NBR1), and autophagy-linked FYVE (ALFY) (Rogov et al., 2014), are involved in autophagy, we cannot exclude the possibility that USP10 promotes protein aggregation and aggresome formation by interacting with other ubiquitin receptors in addition to p62.

USP10 inhibited cell death induced by PI, and the inhibition correlated with aggresome/aggregate-inducing activity (Figures 6 and S5). Accumulating evidence shows that protein oligomers, but not large protein aggregates, have potent cytotoxic activity (Ramdzan et al., 2017). Thus, USP10-induced aggregates/aggresomes might inhibit apoptosis by reducing the amount of ubiquitinated protein oligomers. This mechanism was supported by the following two findings. Although p62 promotes ubiquitinated protein aggregation, large p62 aggregates detected even in USP10-KD cells inhibited apoptosis as efficiently as aggresomes (Figure 6C). Like USP10-KD, p62-KD increased apoptosis induced by treatment with PI (Figures 6E and 6F). However, we could not exclude the possibility that USP10 inhibits apoptosis by promoting degradation of pro-apoptotic protein(s) by aggresome-mediated autophagy.

PI treatment in USP10-WT cells induced p62/HDAC6-double-positive aggresomes, whereas in USP10-KD cells, it induced many small p62-positive/HDAC6-negative aggregates throughout the cytoplasm (Figures 1C and 1D). These results suggested that USP10 functions to stimulate transport of p62 aggregates to the perinuclear aggresome formation site. HDAC6 promotes aggresome formation by stimulating the transport of p62 aggregates to the perinuclear region (Kawaguchi et al., 2003, Yan et al., 2013). Collectively, these results suggest that USP10 stimulates HDAC6-mediated aggresome-inducing activity. Further analysis is required to understand fully how USP10 promotes aggresome formation.

Formation of inclusions containing ubiquitinated proteins is pathognomonic in various neurodegenerative disorders, including PD and Alzheimer disease (Dantuma and Bott, 2014). Accumulating evidence suggests that aggresome-related mechanisms are involved in the formation of the inclusions, including Lewy bodies (Olanow et al., 2004). The findings herein are consistent with this notion. USP10 was detected in the peripheral portion (halo) of Lewy bodies in patients with PD (Figures 7A, 7B, 7F, and 7G). In addition, the amounts of soluble and insoluble USP10 proteins in brain lesions (amygdala) of patients with PD were more than those of the controls (Figure 8A). In cultured cells, USP10 promoted α-synuclein-induced aggresome formation (Figure 3A) and USP10 was often detected at the periphery of α-synuclein-positive aggresomes (Figures S3C and 3C). These results suggest that USP10 may be a factor associated with the formation of Lewy bodies through an aggresome-related mechanism. In contrast, USP10 was not detected in the α-synuclein-positive GCIs in patients with MSA (Figures 7D and 7I), although the GCIs are immunoreactive for several aggresome-marker proteins, including HDAC6 and p62 (Chiba et al., 2012). Thus, the USP10-dependent aggresome-related mechanism is involved in the formation of Lewy bodies, but not GCIs. This difference might be associated with the different physiological expression level of USP10 between neurons and oligodendrocytes.

Based on the data presented above, we propose the following model for USP10 activity in aggresome and pathogenic inclusion formation (Figure 8B). Ubiquitinated proteins generated by various stresses, especially their oligomers, are highly cytotoxic. These ubiquitinated proteins bind to p62 and USP10, and the formed aggregates inhibit proteasome activity to further increase the amount of ubiquitinated proteins. These ubiquitinated proteins then form aggresomes. Thus, USP10 and p62 promote cell survival by reducing the amount of cytotoxic ubiquitinated protein monomers and oligomers. In addition, ubiquitinated proteins in aggresomes are degraded by autophagy or proteasome-mediated degradation, which reduces the amount of ubiquitinated proteins. However, continuous dysfunction of proteasomes and/or autophagy produces pathogenic inclusions such as Lewy bodies. Collectively, we propose that USP10 is an attractive target to control aberrant aggregation and/or cytotoxicity of ubiquitinated proteins in protein aggregation-related diseases including PD.

### Limitations of the Study

We showed that USP10 promotes protein aggregation and aggresome formation by inhibiting the proteasome-mediated degradation of ubiquitinated proteins. It should be noted that ubiquitinated proteins are also degraded by autophagy. In addition, some ubiquitinated proteins such as α-synuclein are secreted from cells. Thus, USP10 might have another activity to promote protein aggregations. Further analysis is required to elucidate how USP10 promotes protein aggregation and aggresome formation.

We proposed that GCI is generated by a USP10-independent mechanism within oligodendrocytes. α-synuclein inclusions have been shown to propagate cell-to-cell by a prion-like mechanism. Thus, GCI might be formed in oligodendrocytes by the endocytosis of extracellular α-synuclein inclusions secreted from other cells such as neurons. Further analysis is required to elucidate how USP10-negative GCI is formed in oligodendrocytes.

## Methods

All methods can be found in the accompanying Transparent Methods supplemental file.

## Supplemental Information

Document S1. Transparent Methods, Figures S1–S5, and Tables S1 and S2
