# Supplementary material for: USP10 Is a Driver of Ubiquitinated Protein Aggregation and Aggresome Formation to Inhibit Apoptosis
Source: iScience. 2018 Nov 5;9:433–50. doi: 10.1016/j.isci.2018.11.006 (PMC6249355; doi:10.1016/j.isci.2018.11.006)
Supplement: Document S1. Transparent Methods, Figures S1–S5, and Tables S1 and S2 [file mmc1.pdf]

ISCI, Volume *9*

Supplemental Information

USP10 Is a Driver of Ubiquitinated Protein Aggregation and Aggresome Formation to Inhibit Apoptosis

Masahiko Takahashi, Hiroki Kitaura, Akiyoshi Kakita, Taichi Kakihana, Yoshinori Katsuragi, Masaaki Nameta, Lu Zhang, Yuriko Iwakura, Hiroyuki Nawa, Masaya Higuchi, Masaaki Komatsu, and Masahiro Fujii

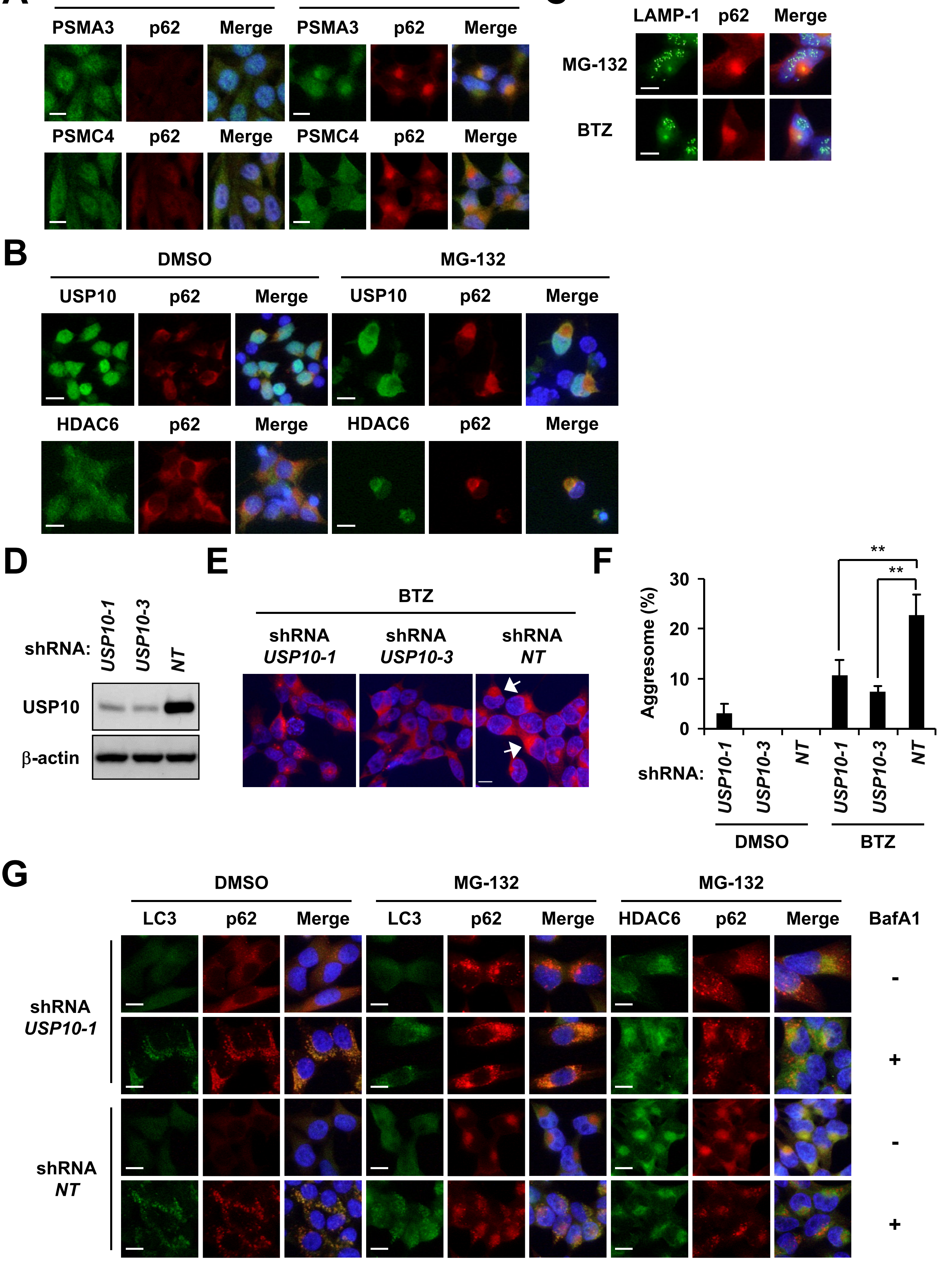
A
DMSO
MG-132
PSMA3
p62
Merge
PSMC4
B
USP10
HDAC6
C
LAMP-1
BTZ
D
shRNA:
USP10-1
USP10-3
NT
USP10
β-actin
E
shRNA
F
Aggresome (%)
30
20
10
0
**
G
LC3
BafA1
-
+

Figure S1, related to Figure 1. USP10 promotes the transport of p62 aggregates to the perinuclear region for aggresome formation

(A) HeLa cells were treated with 5 μM MG-132 or DMSO for 12 hr, and the cells were stained with the anti-p62 (red) antibody and with either the anti-PSMA3 (green) or anti-PSMC4 (green) antibody. Nuclei were counterstained using Hoechst33258 (blue). The bars indicate 10 μm.

(B) Primary neuron-enriched cells prepared from cortical tissues of rat embryo were treated with 2 μM MG-132 or DMSO for 8 hr, and the cells were stained with the anti-p62 (red) antibody together with either the anti-USP10 (green) or anti-HDAC6 (green) antibody. Nuclei were counterstained using Hoechst33258 (blue). The bars indicate 10 μm.

(C) HeLa cells were treated with MG-132 or 1 μM bortezomib (BTZ) for 12 hr, and the cells were stained with anti-LAMP1 (green) and anti-p62 (red) antibodies, and with Hoechst33258 (blue). The bars indicate 10 μm.

(D) 293T cells were infected with lentivirus encoding *USP10* shRNA (*USP10-1* or *USP10-3*) or control siRNA (*NT*). Whole-cell extracts were characterized using western blot analysis (WB) with anti-USP10 and anti-β-actin antibodies.

(E) USP10-KD (*USP10-1* or *USP10-3*) and control (*NT*) 293T cells were treated with 2 μM BTZ or DMSO for 9 hr, and the cells were stained with anti-p62 (red) antibody and Hoechst33258 (blue). Arrows indicate aggresomes (more than 15 μm^2 in size at the perinuclear region with nuclear deformity). The bar indicates 10 μm.

(F) The proportions of cells containing aggresome are presented as the mean ± s.d. ($n = 3$); $^{**}P < 0.01$.

(G) USP10-KD (*USP10-1*) and control (*NT*) HeLa cells were pretreated with 10 nM BafA1 or DMSO and further treated with MG-132 or DMSO for 12 hr. The cells were stained with anti-LC3 (green) and anti-p62 (red) antibodies, and anti-HDAC6 (green) and anti-p62 (red) antibodies. Nuclei were counterstained using Hoechst33258 (blue). The bars indicate 10 μm.

Takahashi et al. Figure S2

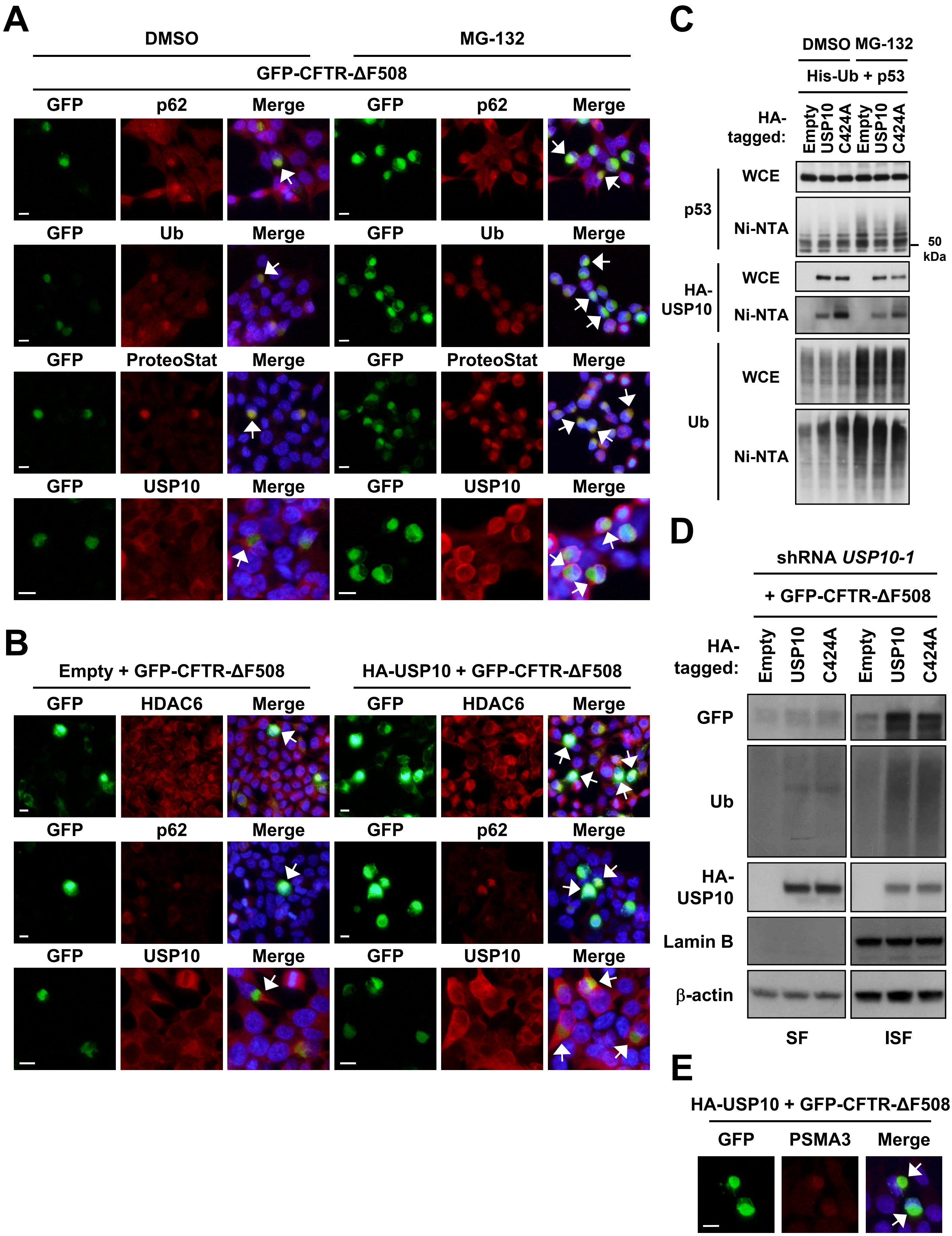

Figure S2, related to Figure 2. CFTR-ΔF508 co-localizes with aggresome markers

(A) HeLa cells were transfected with the GFP-CFTR-ΔF508 plasmid and treated with 5 μM MG-132 or DMSO for 12 hr, and the cells were stained with the anti-p62 antibody (red), anti-ubiquitin (Ub) antibody (red), ProteoStat dye (red) or anti-USP10 (red) antibody and Hoechst33258 (blue). Arrows indicate localization of each aggresome marker at CFTR-ΔF508-induced aggresomes. The bars indicate 10 μm.

(B) HeLa cells were transfected with the HA-USP10 and the GFP-CFTR-ΔF508 plasmids, and these cells were stained with the anti-HDAC6 (red), anti-p62 (red) or USP10 (red) antibody and Hoechst33258 (blue). Arrows indicate localization of each protein at CFTR-ΔF508-induced aggresomes. The bars indicate 10 μm.

(C) HeLa cells were transfected with the His-Ub and p53 plasmids together with HA-USP10 or its deubiquitinase-dead mutant $USP10^{C424A}$ plasmid. Ubiquitinated proteins in cell lysates were collected by Ni-NTA-agarose (Ni-NTA). The whole cell extracts (WCE) and His-ubiquitinated proteins eluted from Ni-NTA agarose were characterized by WB using anti-p53, anti-HA and anti-Ub antibodies.

(D) USP10-KD (*USP10-1*) HeLa cells were transfected with HA-USP10 or its deubiquitinase-dead mutant $USP10^{C424A}$ plasmid together with the GFP-CFTR-ΔF508 plasmid, and NP-40-soluble fractions (SF) and NP40-insoluble fractions (ISF) were characterized by WB using anti-GFP, anti-Ub, anti-HA, anti-Lamin B and anti-β-actin antibodies.

(E) HeLa cells were transfected with the HA-USP10 and GFP-CFTR-ΔF508 plasmids, and these cells were stained with the anti-PSMA3 (red) antibody and Hoechst33258 (blue). Arrows indicate localization of PSMA3 at CFTR-ΔF508-induced aggresomes. The bar indicates 10 μm.

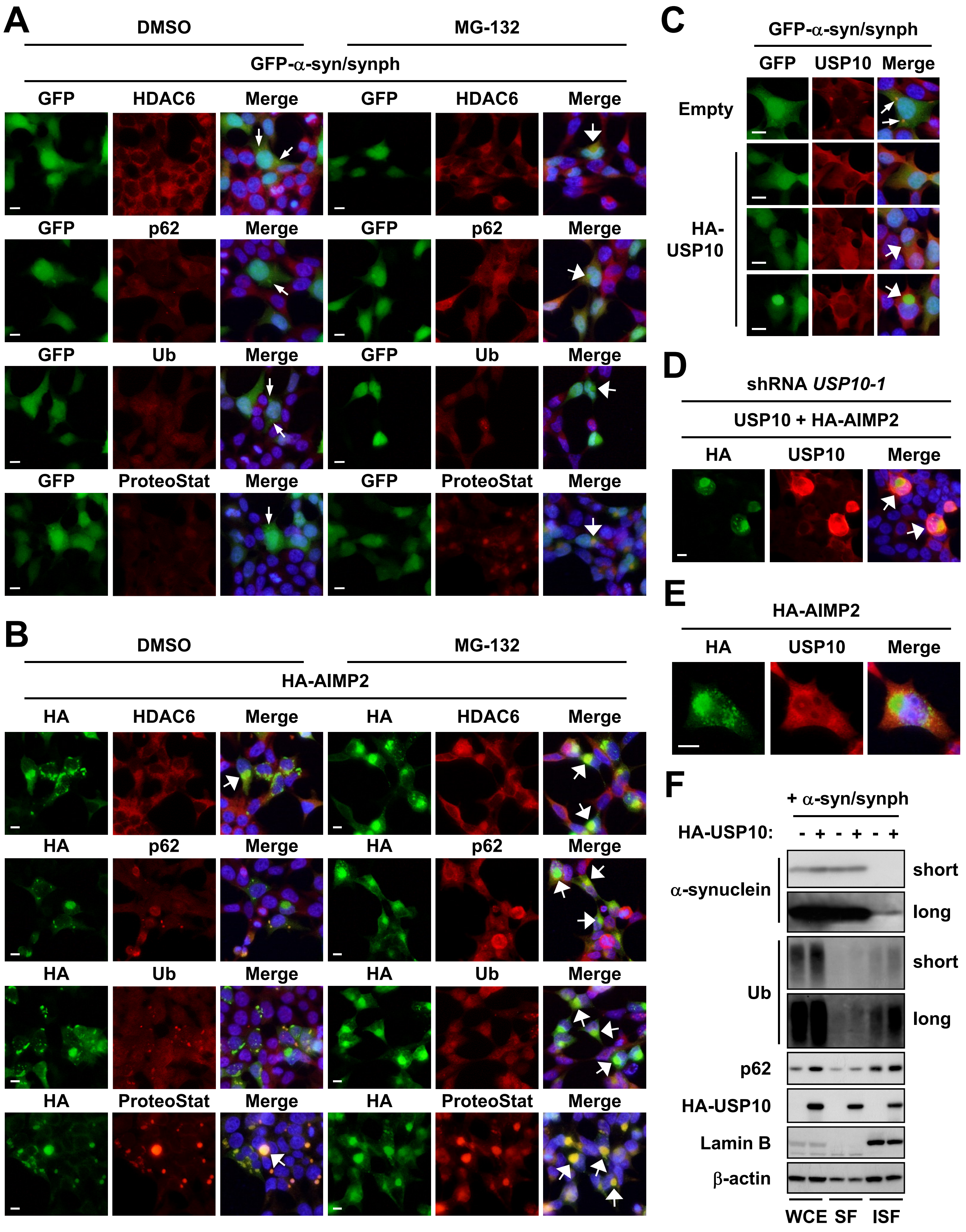
A
DMSO
MG-132
GFP-α-syn/synph
GFP
HDAC6
Merge
p62
Ub
ProteoStat
B
HA-AIMP2
HA
C
GFP-α-syn/synph
GFP
USP10
Merge
Empty
HA-USP10
D
shRNA USP10-1
USP10 + HA-AIMP2
HA
USP10
Merge
E
HA-AIMP2
F
+ α-syn/synph
HA-USP10: - + - + - +
α-synuclein
short
long
Ub
short
long
p62
HA-USP10
Lamin B
β-actin
WCE
SF
ISF

Figure S3, related to Figure 3. α-Synuclein or AIMP2 co-localizes with aggresome markers

(A) HeLa cells were transfected with GFP-α-synuclein (GFP-α-syn) and Myc-synphilin-1 (synph) plasmids and treated with 5 μM MG-132 or DMSO for 12 hr. The cells were stained with the anti-HDAC6 (red), anti-p62 (red) or anti-Ub (red) antibody, or stained with ProteoStat dye (red). Nuclei were counterstained using Hoechst33258 (blue). Small and large arrows indicate α-synuclein-positive aggregates detected under DMSO treatment and α-synuclein-positive aggresome-like aggregates induced by MG-132, respectively. The bars indicate 10 μm.

(B) HeLa cells were transfected with the HA-AIMP2 plasmid and treated with MG-132 or DMSO for 12 hr. The cells were stained with the anti-HA (green) antibody and with either the anti-HDAC6 (red), anti-p62 (red) or anti-Ub (red) antibody, or stained with ProteoStat dye (red). Nuclei were counterstained using Hoechst33258 (blue). Arrows indicate localization of each aggresome marker at HA-AIMP2-induced aggresomes. The bars indicate 10 μm.

(C) HeLa cells were transfected with the HA-USP10 and GFP-α-syn/synph plasmids, and the cells were stained with the anti-USP10 (red) antibody and Hoechst33258 (blue). Small arrows indicate α-synuclein-positive aggregates positive for endogenous USP10, whereas the large arrows indicate α-synuclein-positive aggresomes induced by exogenous HA-USP10 expression. The bars indicate 10 μm.

(D) USP10-KD (*USP10*-1) HeLa cells were transfected with the non-tagged USP10 and the HA-AIMP2 plasmids, and these cells were stained with the anti-HA (green) and anti-USP10 (red) antibodies, and with Hoechst33258 (blue). Arrows indicate HA-AIMP2-positive aggresomes induced by exogenous USP10 expression. The bar indicates 10 μm.

(E) HeLa cells were transfected with the HA-AIMP2 plasmid, and these cells were stained with the anti-HA (green) and anti-USP10 (red) antibodies, and with Hoechst33258 (blue). The bar indicates 10 μm.

(F) HeLa cells were transfected with the HA-USP10, α-synuclein (α-syn) and Myc-synphilin-1 (synph) plasmids, and whole-cell extracts (WCE), NP-40-soluble fractions (SF) and NP-40-insoluble fractions (ISF) were characterized by western blot analysis using anti-α-synuclein, anti-Ub, anti-p62, anti-HA, anti-Lamin B and anti-β-actin antibodies.

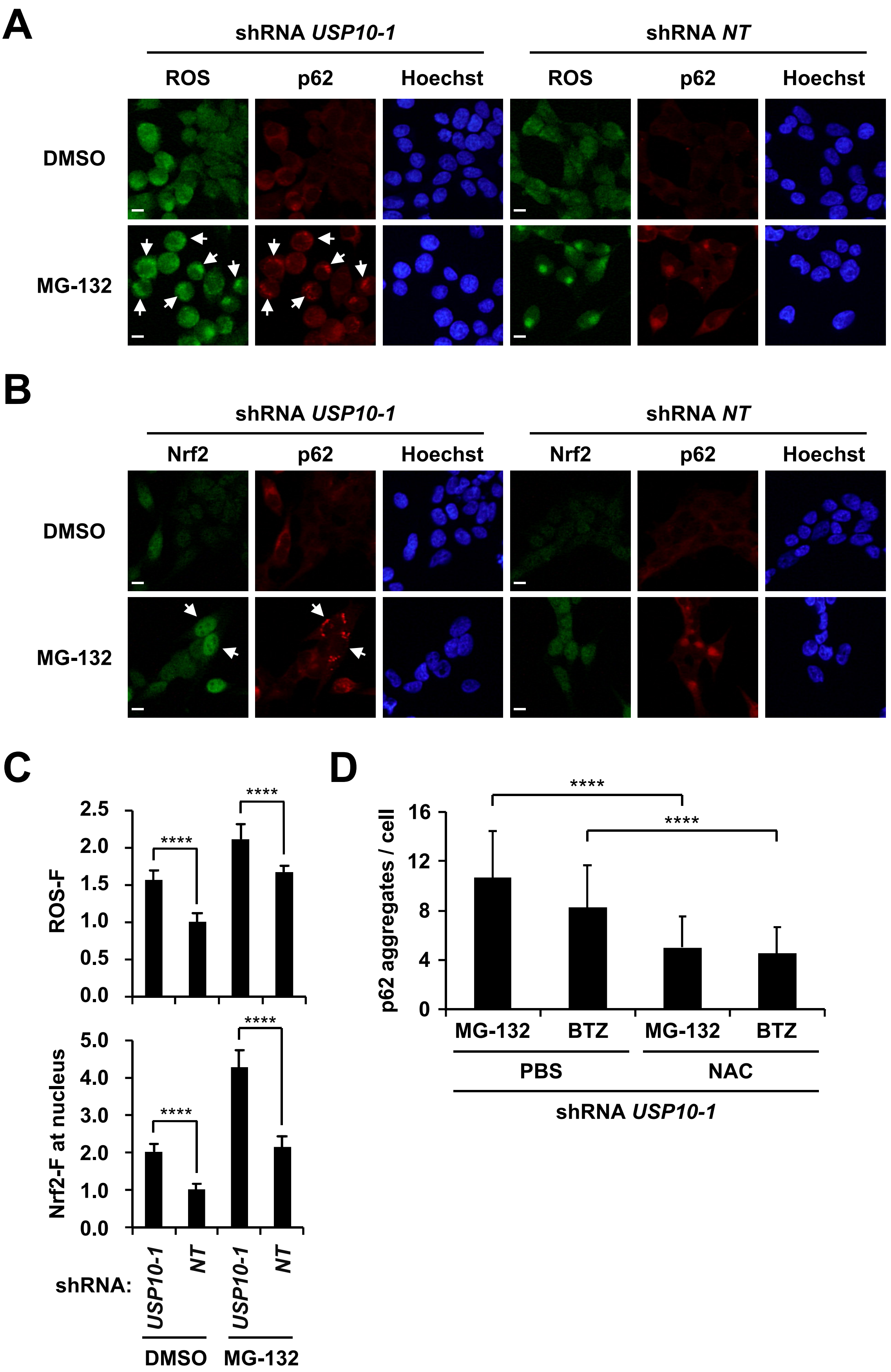
A
shRNA USP10-1
shRNA NT
ROS
p62
Hoechst
DMSO
MG-132
B
Nrf2
C
ROS-F
Nrf2-F at nucleus
shRNA:
USP10-1
NT
DMSO
MG-132
****
D
p62 aggregates / cell
BTZ
PBS
NAC

Figure S4, related to Figure 5. ROS accumulation in USP10-knockdown cells activates p62 aggregation

(A) USP10-KD (*USP10-1*) and control (*NT*) HeLa cells were treated with 5 μM MG-132 or DMSO for 12 hr, and the cells were stained with 5 μM CM-H_2DCFDA (green) and the anti-p62 (red) antibody, and with Hoechst33258 (blue). Arrows indicate cells with a high amount of ROS and p62 aggregates. The bars indicate 10 μm.

(B) The indicated HeLa cells were treated with MG-132 or DMSO for 12 hr, and the cells were stained with the anti-Nrf2 (green) and anti-p62 (red) antibodies, and with Hoechst33258 (blue). Arrows indicate cells with a high amount of Nrf2 in the nucleus and p62 aggregates. The bars indicate 10 μm.

(C) CM-H_2DCFDA fluorescence (ROS-F) or Nrf2 fluorescence at the nucleus (Nrf2-F at nucleus) from panel (A) or (B) are presented as the mean ± s.d. (n = 20); ****$P < 0.0001$.

(D) USP10-KD (*USP10-1*) HeLa cells were pretreated with 20 mM N-acetylcysteine (NAC) or PBS and further treated with MG-132 or 1 μM bortezomib (BTZ) for 12 hr. The cells were stained with the anti-p62 antibody and the numbers of p62 aggregates per cell were counted. The values denote the mean ± s.d. (n = 30); ****$P < 0.0001$.

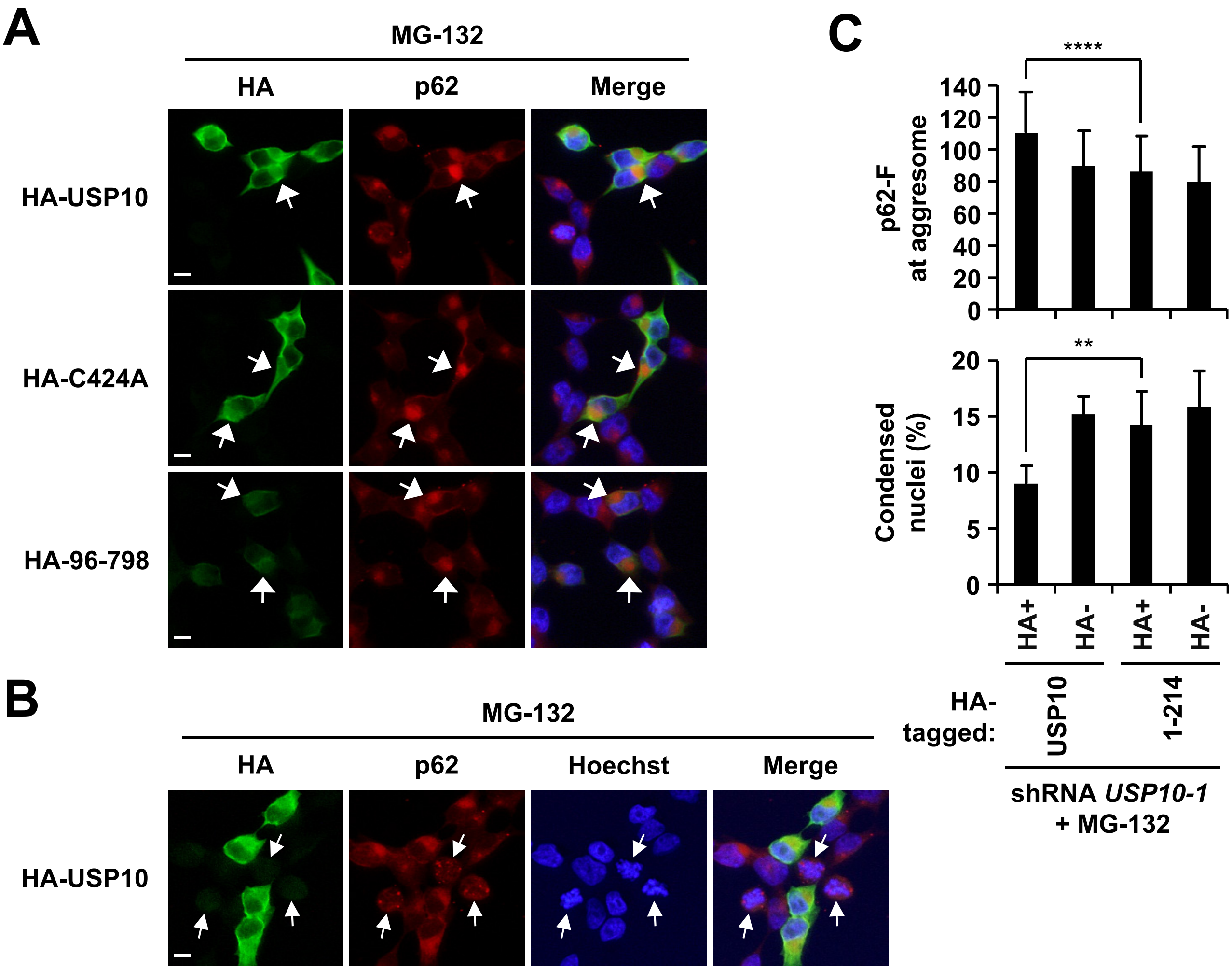

Figure S5, related to Figure 6. Deubiquitinase activity of USP10 is dispensable to inhibit cell death induced by a proteasome inhibitor

(A) USP10-KD (*USP10-1*) HeLa cells were transfected with HA-USP10 or its mutant (USP10^{C424A} or USP10$^{96-798}$) plasmid, and further treated with 5 μM MG-132 for 12 hr. Cells were stained with anti-HA (green) and anti-p62 (red) antibodies, and with Hoechst33258 (blue). The arrows indicate cells showing bright p62-fluorescence because of the overexpression of wild-type USP10, USP10^{C424A} or USP10$^{96-798}$. The bars indicate 10 μm.

(B) Representative image of cell death (condensed nuclei; blue) without the overexpression of exogenous HA-USP10 (green) after MG-132 treatment is shown by the arrows. The bar indicates 10 μm.

(C) USP10-KD (*USP10-1*) HeLa cells were transfected with HA-USP10 or its mutant USP10$^{1-214}$ plasmid and further treated with MG-132 for 12 hr. Cells were analysed in a similar manner to (Figure 6G). The values denote the mean ± s.d. (p62-F at aggresome; $n = 40$, condensed nuclei (%); $n = 6$); $^{**}P < 0.01$; $^{****}P < 0.0001$.

Table S1, related to Figure 7. Clinicopathologic profiles of the patients.

Age at death (yrs)	Sex	Clinical Diagnosis	Pathological Diagnosis
**Control**			
90	M	Myopathy	Myopathy
76	M	Lambert-Eaton synd.	No significant alteration in the brain
51	M	Acute abdomen	No significant alteration in the brain
**PD**			
81	F	PD	PD (DLB neocortical type)
84	F	PD	PD (DLB neocortical type)
79	M	PD	PD (DLB neocortical type)
**MSA**			
68	F	MSA	MSA
82	M	MSA	MSA
71	M	MSA	MSA

PD: Parkinson's disease; DLB: Dementia with Lewy bodies; MSA: Multiple system atrophy

Table S2, related to Figure 8. Clinicopathologic profiles of the patients.

Age at death (yrs)	Sex	Clinical Diagnosis	Pathological Diagnosis
**Control**			
79	F	Spontaneous CSF leaks	Multiple microinfarcts
90	M	Myopathy	Myopathy
**PD**			
79	M	PD	PD (DLB neocortical type)
87	F	PD	PD (DLB neocortical type)
80	M	PD	PD (DLB neocortical type)

PD: Parkinson's disease; DLB: Dementia with Lewy bodies; CSF: cerebrospinal fluid

Transparent Methods

Cell lines and culture condition

HeLa and 293T cells were cultured in Dulbecco's modified Eagle's medium (DMEM) supplemented with 10% heat-inactivated fetal bovine serum (FBS), 4 mM L-glutamine, 50 units/ml penicillin, 50 μg/ml streptomycin and MEM Non-essential amino acid (AA) solution (Thermo Fisher Scientific). Neuro-2a is a mouse neuroblastoma cell line, and cells were cultured in DMEM supplemented with 5% FBS, Opti-MEM (Thermo Fisher Scientific), 50 units/ml penicillin, 50 μg/ml streptomycin and MEM Non-essential AA solution.

Preparation of primary rat neuron-enriched cells

Rat primary neuron-enriched cells were prepared as described previously (Iwakura et al., 2017). Cortical tissues were dissected from rat embryos at embryonic days 16–19 and digested with papain (Worthington Biochemical Corporation). Cells were plated on glass coverslips coated with poly-D-lysine (Sigma-Aldrich) in a 6-well plate (Corning), and cells were cultured in DMEM supplemented with 10% FBS. After overnight culturing, the medium was replaced with serum-free DMEM supplemented with the N-2 Supplement (Gibco) and L-glutamine, and cells were further cultured for 4 d. Cells were then treated with 2 μM MG-132 or DMSO for 8 hr, and characterized by immunostaining.

Sprague-Dawley (SD) rats (Japan SLC, Inc., Shizuoka, Japan) were maintained in the animal care facility of the Niigata University Brain Research Institute. All rats were housed in acrylic cages (24 × 39 × 19.5 cm), and they had food and water ad libitum in a temperature-controlled room (23 ± 2 °C) under a 12-hr light:12-hr dark cycle (light from 8:00 am to 8:00 pm). The Animal Use and Care Committee of Niigata University approved this study and all animal experiments described were carried out in accordance with the institutional guidelines and with those of the National Institutes of Health Guide for the Care and Use of Laboratory Animals (NIH Publications No. 80-23). All efforts were made to minimize discomfort to the rats and the number used.

Reagents and antibodies

The following reagents were purchased from the indicated companies: MG-132 (474790; Calbiochem), bortezomib (B-1408; LC Laboratories), bafilomycin A1 (B1793; Sigma-Aldrich), blasticidin (R21001; Thermo Fisher Scientific), puromycin (P8833; Sigma-Aldrich), N-acetylcysteine (A9165; Sigma-Aldrich) and Hoechst 33258 (H-3569; Molecular Probes). The following antibodies were used in this study: anti-USP10 (A300-901A; Bethyl Laboratories, HPA006731; Sigma-Aldrich), anti-ubiquitin (sc-8017; Santa

Cruz Biotechnology), anti-p62 (PM045; MBL, GP62-C; PROGEN), anti-phospho-p62 (pS351) (PM074; MBL), anti-LC3 (PM036B; MBL), anti-HDAC6 (sc-11420; Santa Cruz Biotechnology), anti-G3BP (611127; BD Transduction Laboratories), anti-dynein intermediate chain (D5167; Sigma-Aldrich), anti-LAMP-1 (L1418; Sigma-Aldrich), anti-HA (H9658; Sigma-Aldrich, 3724; Cell Signaling), anti-GFP (sc-9996; Santa Cruz Biotechnology), anti-Nrf2 (sc-13032; Santa Cruz Biotechnology), anti-PSMA3 (ab109532; Abcam), anti-PSMC4 (sc-166003; Santa Cruz Biotechnology), anti-cleaved caspase-3 (9661; Cell Signaling), anti-p53 (sc-126; Santa Cruz Biotechnology), anti-Lamin B (sc-6217; Santa Cruz Biotechnology), anti-Lamin B1 (sc-374015; Santa Cruz Biotechnology), anti-α-synuclein (S5566; Sigma-Aldrich), anti-phosphorylated α-synuclein (015-25191; Wako), anti-synphilin-1 (sc-365741; Santa Cruz Biotechnology) and anti-β-actin (sc-47778; Santa Cruz Biotechnology).

Plasmids

Expression plasmids encoding wild-type USP10 or mutants (USP10^{C424A}, USP10$^{1–116}$, USP10$^{1–149}$, USP10$^{1–154}$, USP10$^{1–214}$, USP10$^{1–274}$, USP10$^{96–798}$, USP10$^{215–798}$, USP10$^{275–798}$, USP10$^{410–798}$ and USP10$^{594–798}$) with an N-terminal hemagglutinin (HA) epitope tag were generated by subcloning the respective cDNAs into an expression plasmid pCMV-HA (Clontech). The non-tagged USP10 expression plasmid was generated by subcloning USP10 cDNA into the expression plasmid pcDNA3 (Invitrogen). Retroviral expression plasmid pMXs-puro encoding p62 was used for the expression of exogenous p62. pcDNA3 encoding p53 was used for the expression of human p53. Lentiviral expression plasmids encoding wild-type USP10 or its mutants (USP10^{C424A}, USP10$^{96–798}$ and USP10$^{1–214}$) with an N-terminal HA epitope tag were generated by inserting the respective cDNAs into CSII-EF-RfA with a blasticidin resistance gene (Higuchi et al., 2007) using Gateway recombination and following the manufacturer's instructions (Gateway System; Invitrogen). The lentiviral short hairpin RNA (shRNA) plasmid pLKO.1-puro targeting human *USP10* with a puromycin resistant gene was purchased from Sigma-Aldrich. pEGFP-LC3 (Kabeya et al., 2000) (Addgene plasmid #21073) was a gift from Tamotsu Yoshimori. YFP-CL1 (Menendez-Benito et al., 2005) (Addgene plasmid #11950) was a gift from Nico Dantuma.

Plasmid transfection

Plasmids (0.5–1.0 μg) were transfected into HeLa cells (1.5×10^5) on a 6-well plate (Corning) by using the FuGENE 6 reagent according to the manufacturer’s instructions (Roche).

Establishment of stable cell lines by viral transduction

Stable knockdowns of endogenous USP10 in HeLa and 293T cells were carried out by using the lentivirus vectors encoding *USP10* shRNA. HIV-1-based lentiviruses encoding *USP10* shRNA were produced by cotransfection of the three plasmids (pLKO.1-puro: 4.28 μg; pCAG-HIVgp: 2.86 μg; pCMV-VSV-G-RSV-Rev: 2.86 μg) into 293T cells (2×10^6) by using the FuGENE HD reagent according to the manufacturer's instructions (Roche), and the viruses were concentrated with Amicon Ultra-15 units (Millipore) to increase the infectious titer of the virus and infected into HeLa or 293T cells in the presence of 8 μg/μl polybrene. These cells were cultured in the selection medium containing 2 μg/ml puromycin.

Lentiviruses encoding wild-type USP10 or its mutants ($USP10^{C424A}$, $USP10^{96–798}$ and $USP10^{1–214}$) were established using the same method employed for *USP10* shRNA described above, and used for infection into HeLa cells. These cells were cultured in the selection medium containing 5 μg/ml blasticidin.

RNA interference

Small interfering RNAs (siRNA) specific to human *p62* RNA (Oligo ID: HSS113116, HSS113117) and the negative control siRNA (Cat. No. 12935-100) were purchased from Invitrogen. These siRNA (50-100 pmol) were transfected into cells using Lipofectamine RNAiMAX reagents according to the manufacturer's protocol (Invitrogen).

Coimmunoprecipitation assay

The cells (1.0×10^7) on a 10-cm^2 dish (Corning) were lysed with ice-cold NP-40 lysis buffer (1% Nonidet P-40, 25 mM Tris-HCl, pH 7.2, 150 mM NaCl, 1 mM EDTA, 1 mM phenylmethylsulfonyl fluoride, 20 μg/ml aprotinin) and cell lysates were treated with the anti-GFP or anti-p62 antibody. Immune complexes were precipitated by protein G-sepharose beads (GE Healthcare). The beads were washed, boiled in sodium dodecyl sulfate (SDS) lysis buffer (62.5 mM Tris-HCl, pH 6.8, 2% SDS, 10% glycerol, 5% 2-mercaptoethanol and 0.005% bromophenol blue) and then released proteins from the beads were subjected to western blot analysis.

Deubiquitination assay

The cells (4.0×10^5) on a 6-cm^2 dish (Corning) were transfected with the His-ubiquitin and p53 plasmids together with HA-USP10 or its deubiquitinase-dead mutant $USP10^{C424A}$ plasmid. After treatment with 5 μM MG-132 or DMSO for 4 hr, cells were lysed with

buffer A (6 M guanidine-HCl, 0.1 M Na_2HPO_4, 0.1 M NaH_2PO_4, 10 mM imidazole, pH 8.0), and cell lysates were incubated with Ni-NTA-agarose (QIAGEN) at room temperature for 3 hr. This Ni-NTA agarose was sequentially washed with buffer A, buffer A plus B (1:4) and buffer B (25 mM Tris-HCl pH 6.8, 20 mM imidazole), and proteins that bound to the Ni-NTA agarose were eluted by SDS lysis buffer containing 200 mM imidazole. The eluted proteins were then subjected to western blot analysis.

Western blot analysis

The cells were lysed with SDS lysis buffer and cell lysates (20 μg of proteins) were separated by SDS-PAGE, electrophoretically transferred onto a PVDF membrane (Immobilon; Millipore), and treated with the indicated antibodies diluted in Can Get Signal (TOYOBO). Immunoreactive bands were detected with an enhanced chemiluminescence (ECL) detection system (ECL Western Blotting Detection Reagents; GE Healthcare, Pierce™ ECL Plus Western Blotting Substrate; Thermo Fisher Scientific) and were visualized by Amersham Hyperfilm ECL films (Amersham).

Cell fractionation

Three cell lysates (whole-cell extract, soluble fraction and insoluble fraction) were prepared by the following methods. Cultured cells were treated with cold NP-40 lysis buffer, and cell lysates were collected and used as the soluble fraction. The resultant pellet was further treated with the SDS lysis buffer and the lysates were used as the insoluble fraction. Cells were directly treated with the SDS lysis buffer and used as whole-cell extracts. These three lysates were subjected to western blot analysis. Autopsy brain samples from PD patients and the controls were treated with ice-cold Triton X-100 lysis buffer (1% Triton X-100, 50 mM Tris-HCl, pH 7.5, 150 mM NaCl, 1 mM phenylmethylsulfonyl fluoride, 20 μg/ml aprotinin), and the Triton X-100-soluble and Triton X-100-insoluble fractions were collected similarly to the method described above, and two fractions were characterized by western blot analysis.

Immunofluorescence analysis

The cells were cultured on glass coverslips in a 6-well plate, and cells were fixed with 3.7% formaldehyde in PBS and permeabilized by 0.1% Triton X-100 in PBS. Cells were then treated with the primary antibodies, and further incubated with the secondary antibody of either the Alexa488- or Alexa594-conjugated anti-immunoglobulin antibody (anti-mouse, anti-rabbit or anti-guinea pig; Molecular Probes). Cell nuclei were stained with Hoechst 33258. The samples were mounted in Fluoromount/Plus™ (Diagnostic

Biosystems) and the images were analysed with a fluorescence microscope (BZ-8000; KEYENCE).

Microscope analysis

Cells with one large HDAC6/p62-double-positive aggregate (more than 15 μm^2 in size) at the perinuclear region with nuclear deformity were counted as aggresome-positive cells. Cells with multiple small HDAC6-negative/p62-positive aggregates (less than 10 μm^2 in size) were counted as p62-aggregate-positive cells. To measure cells with p62/HDAC6-positive aggresome or p62-positive/HDAC6-negative aggregates, 300 cells in random fields from three coverslips were analysed by staining with anti-HDAC6 and anti-p62 antibodies. Percentages of cells with p62/HDAC6-positive aggresome or p62-positive/HDAC6-negative aggregates were calculated as the ratio of aggresome-positive or p62-aggregates-positive cells relative to total cells. To measure cells with aggresome induced by GFP-CFTR-ΔF508, GFP-α-synuclein/synphilin-1 or HA-AIMP2, at least 300 cells in random fields from three or four coverslips were counted. One large GFP- or HA-positive aggregate (more than 15 μm^2 in size) at the perinuclear region with nuclear deformity was evaluated as an aggresome. Percentages of cells with GFP- or HA-positive aggresome were calculated as the ratio of aggresome-positive cells relative to total cells. To measure the size of p62-aggregate (p62 aggregate (μm^2)), cells were transfected with the p62 expression plasmid and the sizes of p62-aggregates were quantified from 30 randomly selected p62-aggregates using the fluorescent analysis software package (BZ-II analyser; KEYENCE). The number of p62-aggregates per cell was counted from 30 randomly selected cells treated with proteasome inhibitors in the presence or absence of N-acetylcysteine. To measure p62 fluorescence intensity at the aggresome (p62-F at aggresome) or Nrf2 fluorescence intensity at the nucleus (Nrf2-F at nucleus), these fluorescence intensities were measured from 20 randomly selected cells using the BZ-II analyser. Detection of ROS was performed as described previously (Takahashi et al., 2013). Briefly, cells were incubated with 5 μM of 5-(and-6)-chloromethyl-2'7'-dichlorodihydrofluorescein diacetate, acetyl ester (CM-H_2DCFDA; Molecular Probes) for 5 min at 37 °C. The cells were fixed with 3.7% formaldehyde in PBS and the CM-H_2DCFDA-fluorescence intensity per cell was quantified using BZ-II analyser. Twenty cells in random fields were analysed and the data are presented as the mean fluorescence intensity (ROS-F). Two methods were used to measure the level of apoptosis. First, apoptotic cells on coverslips were visualized by an anti-cleaved caspase-3 antibody and then stained cells were counted under a fluorescence microscope. Next, cell nuclei were stained with Hoechst33258 and cells with condensed nuclei were counted under a

fluorescence microscope. Three hundred cells in random fields from three coverslips were analysed (cleaved caspase-3 (%) or condensed nuclei (%)).

Detection of protein aggregates by the ProteoStat dye

The ProteoStat Aggresome Detection Kit (Enzo Life Sciences, NY, USA) was used according to the manufacturer's protocol to detect protein aggregates.

Pathological analysis

The study was performed with the approval of the ethics committees of Niigata University. The autopsied brain tissues of patients with PD (*n* = 3), MSA (*n* = 3) and controls (*n* = 3) were used. Formalin-fixed, paraffin-embedded sections, 4-μm-thick, were immunostained using a rabbit polyclonal antibody against USP10 (HPA006731; Sigma-Aldrich) and a mouse monoclonal antibody against phosphorylated α-synuclein (015-25191; Wako). The deparaffinized sections were incubated with formic acid for 5 min and autoclaved for 10 min at 121 °C in citrate buffer. Immunolabeling was detected using the peroxidase-polymer–based method using the Histofine Simple Stain MAX-PO kit (Nichirei Biosciences) and visualized with a diaminobenzidine/H_2O_2 solution. Counterstaining was carried out with hematoxylin. For immunofluorescent imaging, primary antibodies were detected by Alexa488- or Alexa568-conjugated anti-immunoglobulin secondary antibodies (appropriately anti-mouse or anti-rabbit; Molecular Probes). These signals were observed under a confocal microscope (FV3000RS; Olympus).

Statistical analysis

Data were analysed with the Student's *t*-test or one-way ANOVA followed by Tukey's Multiple Comparisons Test using Prism7 software (GraphPad) and are presented as the mean ± sd.

Supplemental References

Higuchi, M., Tsubata, C., Kondo, R., Yoshida, S., Takahashi, M., Oie, M., Tanaka, Y., Mahieux, R., Matsuoka, M., and Fujii, M. (2007). Cooperation of NF-kappaB2/p100 activation and the PDZ domain binding motif signal in human T-cell leukemia virus type 1 (HTLV-1) Tax1 but not HTLV-2 Tax2 is crucial for interleukin-2-independent growth transformation of a T-cell line. Journal of virology 81, 11900-11907.

Iwakura, Y., Wang, R., Inamura, N., Araki, K., Higashiyama, S., Takei, N., and Nawa, H. (2017). Glutamate-dependent ectodomain shedding of neuregulin-1 type II precursors in rat forebrain neurons. PloS one 12, e0174780.

Kabeya, Y., Mizushima, N., Ueno, T., Yamamoto, A., Kirisako, T., Noda, T., Kominami, E., Ohsumi, Y., and Yoshimori, T. (2000). LC3, a mammalian homologue of yeast Apg8p, is localized in autophagosome membranes after processing. The EMBO journal 19, 5720-5728.

Menendez-Benito, V., Verhoef, L.G., Masucci, M.G., and Dantuma, N.P. (2005). Endoplasmic reticulum stress compromises the ubiquitin-proteasome system. Human molecular genetics 14, 2787-2799.

Takahashi, M., Higuchi, M., Matsuki, H., Yoshita, M., Ohsawa, T., Oie, M., and Fujii, M. (2013). Stress granules inhibit apoptosis by reducing reactive oxygen species production. Mol Cell Biol 33, 815-829.
